# Isometric Exercise Training and Arterial Hypertension: An Updated Review

**DOI:** 10.1007/s40279-024-02036-x

**Published:** 2024-05-19

**Authors:** Jamie J. Edwards, Damian A. Coleman, Raphael M. Ritti-Dias, Breno Q. Farah, David J. Stensel, Sam J. E. Lucas, Philip J. Millar, Ben D. H. Gordon, Véronique Cornelissen, Neil A. Smart, Debra J. Carlson, Cheri McGowan, Ian Swaine, Linda S. Pescatello, Reuben Howden, Stewart Bruce-Low, Christopher K. T. Farmer, Paul Leeson, Rajan Sharma, Jamie M. O’Driscoll

**Affiliations:** 1 School of Psychology and Life Sciences, Canterbury Christ Church University, Kent, CT1 1QU UK; 2 Graduate Program in Rehabilitation Sciences, University Nove de Julho, São Paulo, Brazil; 3 Department of Physical Education, Universidade Federal Rural de Pernambuco, Recife, Brazil; 4 National Centre for Sport and Exercise Medicine, School of Sport, Exercise and Health Sciences, Loughborough University, Loughborough, UK; 5 NIHR Leicester Biomedical Research Centre, University Hospitals of Leicester NHS Trust and the University of Leicester, Leicester, UK; 6 Faculty of Sport Sciences, Waseda University, Tokyo, Japan; 7 Department of Sports Science and Physical Education, The Chinese University of Hong Kong, Hong Kong, China; 8 School of Sport, Exercise and Rehabilitation Sciences, University of Birmingham, Birmingham, UK; 9 Human Cardiovascular Physiology Laboratory, Department of Human Health and Nutritional Sciences, College of Biological Sciences, University of Guelph, Guelph, ON Canada; 10 Department of Health and Human Development, University of Pittsburgh, Pittsburgh, PA USA; 11 Department of Rehabilitation Sciences, KU Leuven, Leuven, Belgium; 12 School of Science and Technology, University of New England, Armidale, NSW Australia; 13 School of Health, Medical and Applied Sciences, CQ University, North Rockhampton, QLD Australia; 14 Department of Kinesiology, University of Windsor, Windsor, ON Canada; 15 Sport Science, University of Greenwich, London, UK; 16 Department of Kinesiology, University of Connecticut, Storrs, CT 06269 USA; 17 Department of Applied Physiology, Health and Clinical Sciences, UNC Charlotte, Charlotte, NC 28223 USA; 18 Department of Applied Sport and Exercise Science, University of East London, London, UK; 19 Centre for Health Services Studies, University of Kent, Canterbury, UK; 20 Oxford Clinical Cardiovascular Research Facility, Department of Cardiovascular Medicine, University of Oxford, Oxford, UK; 21 Department of Cardiology, St George’s University Hospitals NHS Foundation Trust, Blackshaw Road, Tooting, London, SW17 0QT UK

## Abstract

Hypertension is recognised as a leading attributable risk factor for cardiovascular disease and premature mortality. Global initiatives towards the prevention and treatment of arterial hypertension are centred around non-pharmacological lifestyle modification. Exercise recommendations differ between professional and scientific organisations, but are generally unanimous on the primary role of traditional aerobic and dynamic resistance exercise. In recent years, isometric exercise training (IET) has emerged as an effective novel exercise intervention with consistent evidence of reductions in blood pressure (BP) superior to that reported from traditional guideline-recommended exercise modes. Despite a wealth of emerging new data and endorsement by select governing bodies, IET remains underutilised and is not widely prescribed in clinical practice. This expert-informed review critically examines the role of IET as a potential adjuvant tool in the future clinical management of BP. We explore the efficacy, prescription protocols, evidence quality and certainty, acute cardiovascular stimulus, and physiological mechanisms underpinning its anti-hypertensive effects. We end the review with take-home suggestions regarding the direction of future IET research.

## Key Points

**Table:** 

This work presents an expert-informed review on the role of isometric exercise training in the prevention and treatment of arterial hypertension, covering the efficacy, prescription protocols, evidence quality and certainty, acute cardiovascular stimulus, and physiological mechanisms underpinning its anti-hypertensive effect.
Data from prospective randomised controlled trials and meta-analyses indicate that isometric exercise training is capable of producing blood pressure reductions greater than that observed following the currently recommended exercise guidelines and possibly even greater, or at least similar to that of standard anti-hypertensive monotherapy.
Several domains within the literature require further empirical attention; however, current evidence supports the clinical implementation of IET for the management of blood pressure.

## Introduction

Non-communicable diseases are responsible for an estimated 73% of all deaths globally, of which cardiovascular disease (CVD) remains the principal culprit [1]. Approximately 31% of all deaths are directly attributable to CVD, making it the leading cause of mortality worldwide [1, 2]. Specifically, ischaemic heart disease and cerebrovascular accidents collectively account for 84.9% of all CVD deaths, with the remaining sum of mortality a consequence of other cardiac or vascular pathology, such as calcific valvular disease and peripheral vascular disease [1, 3].

**Fig. 1:**
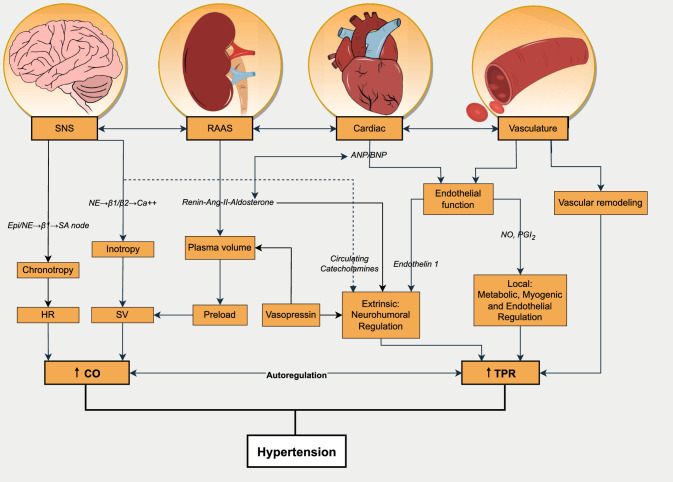
Pathophysiological mechanisms of hypertension. *Ang* angiotensin, *ANP* atrial natriuretic peptide, *BNP* brain natriuretic peptide, *CA* calcium, *CO* cardiac output, *Epi* epinephrine, *HR* heart rate, *NE* norepinephrine, *NO* nitric oxide, *PGI* prostacyclin, *RAAS* renin–angiotensin–aldosterone system, *SNS* sympathetic nervous system, *SV* stroke volume, *TPR* total peripheral resistance

The underlying pathophysiology responsible for the development of CVD is dependent upon the complex interplay of a number of variables, many of which are unclear, that intricately interact throughout the course of human life. Through generations of empirical investigation, risk factors that contribute to the progression of CVD have been established and defined as non-modifiable (such as sex, age, and ethnicity) or modifiable (such as body mass index, smoking status, alcohol consumption), depending upon the capacity for external influence. Elevated blood pressure (BP), which is clinically termed hypertension (HTN), is recognised as the leading attributable risk factor for both CVD and mortality [4] (Fig. 1).

Briefly, BP can be defined as the measurement of hydraulic force exerted on the arterial walls by oxygenated blood in the systemic circulation [5]. Systolic BP (sBP) refers to the arterial pressure during myocardial contraction, while diastolic BP (dBP) describes the state of pressure during the relaxation phase of a cardiac contraction. The current classification of BP varies depending on the guidelines adopted. Guidelines provided by the National Institute for Health and Care Excellence (NICE) and the European Society of Cardiology/European Society of Hypertension (ESC/ESH) determine a diagnosis of HTN at ≥ 140 mmHg sBP, and/or ≥ 90 mmHg dBP [6, 7]. However, the current American Heart Association/American College of Cardiology (AHA/ACC) guidelines set a lower treatment threshold for HTN diagnosis at ≥ 130 mmHg sBP and/or ≥ 80 mmHg dBP [8]. Regardless of this diagnostic confliction, it is uniformly accepted that sBP and dBP values of < 120 mmHg and < 80 mmHg are optimal, and increasing pressure beyond this threshold is linearly associated with an escalated risk of CVD [9, 10]. Specifically, the risk of CVD has been reported to double for every increase in sBP by 20 mmHg, with a more recent analysis reporting a 13% increase in risk of mortality for every 10 mmHg increase in sBP [9, 11]. The SPRINT trial of 9361 patients demonstrated that targeting treatment to a sBP of < 120 mmHg as opposed to the standard practice of < 140 mmHg resulted in lower rates of fatal and nonfatal major cardiovascular events and all-cause mortality [12].

HTN is estimated to affect 1.13 billion people globally, and due to its asymptomatic nature, this figure may be an underestimate [13, 14]. Given this immense global prevalence and the sequelae of HTN, approaches to BP management have been extensively studied over the past half century. With this, a plethora of anti-hypertensive pharmacological treatment options have been established as highly efficacious in reducing BP and consequently improving patient outcomes [15, 16]. As a result, the widespread clinical application of pharmacotherapy in the management of BP is vast. For example, survey research shows that 77.3% of Americans diagnosed with HTN are medicated [17], while the number of adults receiving pharmacological treatment for BP increased by 50% from 2006 to 2016 in England [18]. Despite such prevalence, there are substantial limitations associated with medication for HTN, which are often underestimated in the clinical practice, including adverse effects, economic burden and the risk of prescription errors resulting in unintended consequences [19, 20]. Furthermore, adherence to anti-hypertensive medication is typically reported at < 50% 1 year following initial prescription [21]. Poor adherence to BP medication is associated with a 75% increase in the risk of all-cause mortality [22]. Once a patient is first prescribed medication, they are also likely to remain dependent for life, marking an important treatment crossroad for clinicians [23].

Therefore, establishing effective, adherable, non-pharmacological approaches may prove pivotal in tackling the global HTN crisis. Non-pharmacological treatment includes weight loss, smoking cessation, healthy diet, reduced intake of dietary sodium, enhanced intake of dietary potassium, moderation in alcohol and physical activity [7]. This review critically examines the role of isometric exercise training (IET) as a potential adjuvant tool in the future clinical management of BP. We explore the efficacy, acute cardiovascular stimulus, and physiological mechanisms underpinning its anti-hypertensive impact. Despite the BP-lowering benefits and endorsement by select governing bodies, IET is not widely promoted or prescribed in clinical practice. We end the review with take-home suggestions regarding the direction of future IET research.

## Isometric Exercise Training: Current Evidence

Isometric exercise refers to a sustained muscular contraction in which the length of the muscle does not change. In recent years, many research trials have investigated the effects of IET on BP, employing various protocols and modes of application. While no single benchmark protocol has been established, the majority of IET research has utilised a handgrip (dynamometer) protocol, generally performed at 30% of the participant’s maximal voluntary contraction (MVC) [24–26]. Conversely, few have investigated bilateral leg extension IET, typically applied at an intensity of 20% MVC or 85% HR_peak_ via an isokinetic dynamometer [27, 28]. Finally, more recent work has demonstrated the efficacy of IET employed in the variation of a wall squat requiring an incremental test to establish individualised intensity thresholds of 95% HR_peak_ [29, 30]. Regardless of the approach, the most commonly studied protocols require a time commitment of approximately 11–20 min per session. This is significantly less than that of other more conventional exercise modes, with aerobic and dynamic resistance training sessions typically ranging from 30 min to > 1 h. In addition to its time efficiency, the appeal of IET surrounds its practicality with minimal equipment requirements, wide versatility in applicable environments (e.g., home-based and work environments), and general accessibility. The wall squat protocol can be applied with no equipment and the handgrip protocol only requires a commercially available dynamometer. The leg extension, however, is considerably less accessible, generally requiring a costly isokinetic dynamometer or equivalent and is utilised the least of all applications in the IET field.

### Evidence

Tables 1 and 2 provide all current meta-analysis and randomised controlled trial (RCT) data recognised in the development of this review. These studies were predominantly identified via an update of a systematic search which has been detailed previously [31]. This search was performed in PubMed (MEDLINE), the Cochrane Library and SPORTDiscus and included MeSH terms, key words and word variants for ‘isometric exercise training’, ‘static contraction’, ‘exercise training’, ‘blood pressure’ and ‘hypertension’. Individual RCTs in this review were also found through previous meta-analysis research in this area (Table 1). To most effectively represent only valid and rigorous evidence, Table 2 exclusively includes only RCTs published from January 1, 2000, to April 1, 2023, that have investigated the pre- and post-BP changes following any IET intervention.

**Table 1:** Systematic review and meta-analytic data on the effects of isometric exercise training on resting blood pressure

Systematic review with meta-analysis	Analysis details	Inclusion/exclusion criteria	Outcome	Subgroup outcome
Owen et al. [110]	Published February 2010 5 trials 122 participants	RCTs published ≤ 2009	sBP: − 10.4 mmHg dBP: − 6.7 mmHg	N/A
Kelly and Kelly [117]	Published March 2010 3 trials 81 participants	RCTs published from 1971 to Feb 2009 ≥ 4-week intervention	sBP: − 13.4 mmHg dBP: − 7.8 mmHg	N/A
Cornelissen et al. [118]	Published September 2011 3 trials 82 participants	RCTs published ≤ June 2010	sBP: − 13.5 mmHg dBP: − 7.8 mmHg	N/A
Cornelissen & Smart. [91]	Published February 2013 5 trials 150 participants	RCTs published from 1976 to Feb 2012 ≥ 4-week intervention	sBP: − 10.9 mmHg dBP: − 6.2 mmHg	N/A
Carlson et al. [102]	Published March 2014 9 trials 223 participants	RCTs published from 1966 to July 2013 ≥ 4-week intervention	sBP: − 6.77 mmHg dBP: − 3.96 mmHg mBP: − 3.94 mmHg	N/A
Jin et al. [111]	Published January 2017 6 trials 157 participants	RCTs ≤ Nov 2014 Handgrip studies only ≥ 4-week intervention	sBP: − 8.33 mmHg dBP: − 3.93 mmHg	*HTN:* sBP and dBP significantly decreased in NTN, pre-HTN and HTN, although greatest reductions in pre-HTN
Inder et al. [112]	Published October 2016 11 trials 302 participants	RCTs and cross-over trials published from 1966 to Jan 2015 ≥ 2-week intervention	sBP: − 5.20 mmHg dBP: − 3.91 mmHg mBP: − 3.33 mmHg	*Sex:* greater reduction in males *Age:* greater reductions in older participants (≥ 45 y) *Duration:* Greater reductions in interventions ≥ 8 wk *HTN:* Greater reductions in hypertensive participants *Unilateral:* greater reductions in unilateral than bilateral IET *Upper vs lower:* greater reductions in arm vs lower limb IET
Loaiza-Betancur et al. [119]	Published March 2020 6 trials 139 participants	RCTs published < 2016 Normotensive participants only (< 120 mmHg and < 80 mmHg)	sBP: − 2.83 mmHg dBP: − 2.73 mmHg mBP: − 3.07 mmHg	N/A
Loaiza-Betancur et al. [96]	Published August 2020 11 trials 311 participants	RCTs published < Jan 2018 ≤ 50% MVC intensity	sBP: − 5.43 mmHg dBP: − 2.41 mmHg mBP: − 1.28 mmHg	*HTN:* mBP significantly decreased in pre-HTN, but sBP and dBP did not. sBP significantly decreased in HTN, but not dBP or mBP *BMI:* sBP but not dBP significantly decreased in healthy BMI participants. No significant reductions were found in overweight or obese patients separately *Age:* sBP significantly decreased in ≥ 50-year-olds, but not dBP or mBP. BP did not significantly decrease in < 45-year-olds *Medication:* patients on anti-hypertensive medication had significantly decreased dBP, but not sBP or mBP
López-Valenciano et al. [113]	Published July 2019 16 trials 492 participants	RCTs published < Jan 2018 ≥ 2-week intervention	sBP: − 5.23 mmHg dBP: − 1.64 mmHg mBP: − 2.9 mmHg	*HTN*: greater reductions in normotensive vs hypertensives participants *Other:* no significant effects of sex, age, clinical status, intervention duration, mode or intensity
Naci et al. [90]	Published July 2019 12 trials	RCTs published < Sep 2018	sBP: − 5.65 mmHg	N/A
Hansford et al. [114]	Published August 2021 24 trials 1143 participants	RCTs published < Aug 2020 ≥ 3-week intervention	sBP: − 6.97 mmHg dBP: − 3.86 mmHg	*HTN:* similar statistically significant sBP reductions in pre-HTN and HTN. Greater dBP reductions in HTN than pre-HTN, although both statistically significant *Mode:* greater sBP reductions in leg than handgrip IET, although both statistically significant. Greater dBP reductions in handgrip, with no significant change in leg IET
Edwards et al. [103]	Published December 2021 18 trials 672 participants	RCTs published from Jan 2000 to Sep 2020 Intervention duration 2–12 weeks	sBP: − 8.50 mmHg dBP: − 4.07 mmHg mBP: − 6.46 mmHg	*Upper vs lower body IET:* no significant difference No significance moderator effects for hypertension diagnosis, medication status or intervention duration
Edwards et al. [120]	Published August 2022 18 trials 628 participants	RCTs published from 2000 to Dec 2021 Intervention duration 2–12 weeks Reported at least 1 mechanistic parameter alongside the primary BP change	sBP: − 9.34 mmHg dBP: − 4.30 mmHg mBP: − 5.21 mmHg	*Mode:* non-statistically significant differences (although clinically significant) between wall squat, leg extension and handgrip

*BMI* body mass index, *dBP* diastolic blood pressure, *HTN* hypertension, *IET* isometric exercise training, *mBP* mean blood pressure, *NTN* normotension, *pre-HTN* pre-hypertension, *RCT* randomised controlled trial, *sBP* systolic blood pressure

**Table 2:** Prospective randomised controlled trials investigating the effects of isometric exercise training on resting blood pressure

Randomised controlled trial	Country	Duration (weeks)	Participants	Hypertension (according to baseline BP or medication status)	Included medication	Withdrawal (n. of participants)	Training frequency	Exercise mode	Exercise training characteristics	Pre-IET mean sBP/dBP (mmHg)	Post-IET mean sBP/dBP (mmHg)
Baddeley-White et al. (2019) [77]	UK	4	*N* = 23 (43% female)	NTN	No	None	3 × per week	Isoball rugby handgrip/zona plus handgrip	4 × 2 min, bilateral (alternating hands), 1-min rest interval, 30% MVC (*n* = 7 isoball, *n* = 8 zona, *n* = 8 control)	Isoball 129.3/70 mmHg Zona Plus 125.5/71.6 mmHg	Isoball 119.9/65.7 mmHg Zona Plus 114.5/66.6 mmHg
Badrov et al. (2013) [67]	Canada	8	*N* = 32 (100% female)	NTN	No	IET 1 Control 3	3/5 × per week	Handgrip	4 × 2 min, unilateral 4-min rest intervals, 30% MVC (*n* = 12 3 × per week, *n* = 11 5 × per week, *n* = 9 control)	3 days p/w 94/57 mmHg 5 days p/w 97/57 mmHg	3 days p/w 88/54 mmHg 5 days p/w 91/57 mmHg
Badrov et al. (2013) [180]	Canada	10	*N* = 24 (46% female)	HTN	Yes	None	3 × per week	Handgrip	4 × 2 min, bilateral (alternating hands), 1-min rest interval, 30% MVC (*n* = 12 IET, *n* = 12 control)	129/72 mmHg	121/67 mmHg
Baross et al. (2012) [63]	UK	8	*N* = 30 (100% male)	Pre-HTN	No	None	3 × per week	Leg extension (bilateral)	4 × 2 min, 2-min rest intervals, 14% MVC (*n* = 10 at 85%HR_peak_, *n* = 10 at 75%HR_peak_, *n* = 10 control)	High int 138.7/78.2 mmHg Low 137.3/78.3 mmHg	High int 127.9/76.6 mmHg Low 136.5/79.4 mmHg
Baross et al. (2013) [28]	UK	8	*N* = 20 (100% male)	Pre-HTN	No	None	3 × per week	Leg extension (bilateral)	4 × 2 min, 2-min rest intervals (85%HR_peak_ *n* = 10 exercise group, *n* = 10 control)	139/85 mmHg	128/83 mmHg
Baross et al. (2022) [45]	UK	8	*N* = 25 (36% female)	NTN	No	None	3 × per week	Leg extension (bilateral)	4 × 2 min, 2-min rest intervals (*n* = 13 20% MVC exercise group, *n* = 12 control)	123/70.3 mmHg	118/68.2 mmHg
Rodrigues et al. (2019) [76]	Brazil	12	*N* = 72 (67% female)	HTN	Yes	IET 31 Control 8	3 × per week	Handgrip	4 × 2 min, bilateral (alternating hands), 1-min rest interval (*n* = 17 30% MVC, *n* = 16 control)	135/73 mmHg	121/66 mmHg
Cohen et al. (2022) [68]	Colombia	12	*N* = 77 (33% female)	Pre-HTN	No	None	3 × per week	Handgrip or wall squat	Handgrip: 4 × 2 min, 2-min rest intervals (*n* = 28 30% MVC) Wall squat: 4 × 2 min, 2-min rest intervals (*n* = 27) 22 controls	Handgrip: 140/86.7 mmHg Wall squat: 141.2/87 mmHg	Handgrip: 128.8/82.7 mmHg Wall squat: 128.3/82.9 mmHg
Carlson et al. (2016) [25]	Australia	8	*N* = 40 (62.5% female)	HTN	Yes	IET 2	3 × per week	Handgrip	4 × 2 min, unilateral 1-min rest intervals (*n* = 18 at 30% MVC, *n* = 20 5% MVC exercise control)	136/77 mmHg	129/75 mmHg
Correia et al. (2020) [72]	Brazil	8	*N* = 102 (sex unknown)	HTN	Yes	IET 21 Control 2	3 × per week	Handgrip	4 × 2 min, unilateral, 4-min rest intervals (*n* = 29 30% MVC, *n* = 50 control)	142/75 mmHg	136/72 mmHg
Decaux et al. (2021) [65]	UK	4	*N* = 20 (50% female)	Pre-HTN	No	None	3 × per week	Wall squat	4 × 2 min, 2-min rest intervals (*n* = 10 95% HR_peak_, *n* = 10 control)	131/79.7 mmHg	115.8/75.1 mmHg
Farah et al. (2018) [24]	USA	12	*N* = 72 (75% female) 48 > 50 years	HTN	Yes	Home IET 6 Supervised IET 10 Control 8	3 × per week	Handgrip	4 × 2 min, bilateral (alternating hands), 1-min rest intervals (*n* = 18 at 30% MVC home based, *n* = 14 30% MVC supervised, *n* = 16 control)	Home 130/73 mmHg Supervised 132/71 mmHg	Home 126/71 mmHg Supervised 120/66 mmHg
Fecchio et al. (2023) [85]	Brazil	10	*N* = 35 (100% male)	Pre-HTN	Yes	IET 3 Control 1	3 × per week	Handgrip	4 × 2 min, unilateral, 1-min rest intervals (*n* = 16 control)	128/87 mmHg	125/86 mmHg
Gill et al. (2014) [87]	USA	3	*N* = 40 (71% female)	NTN	No	IET-20: 2 IET-30: 1 Control: 2	3 × per week	Leg extension (bilateral)	4 × 2 min, 3-min rest intervals (*n* = 8 20%EMG_peak_, *n* = 9 30%EMG_peak_ *n* = 18 control)	20%EMG_peak_ 116.4/68.7 mmHg 30%EMG_peak_ 110.4/62.1 mmHg	20%EMG_peak_ 113.7/66.7 mmHg 30%EMG_peak_ 107.4/58.2 mmHg
Gordon et al. (2018) [84]	USA	12	*N* = 22 (sex unknown) 22 < 50 years	Pre-HTN/HTN	Yes	None	3 × per week	Handgrip	4 × 2 min, unilateral, 1-min rest intervals (*n* = 5 30% MVC home-based, *n* = 8 30% MVC lab-based, *n* = 9 control)	Home 137.7/88.4 mmHg Lab 137.6/87.1 mmHg	Home 128/81.6 mmHg Lab 128.5/84.3 mmHg
Javidi et al. (2022) [66]	Iran	8	*N* = 39 (100% male)	HTN	No	IHG-60: 3 IHG-30: 2 Control: 1	3 × per week	Handgrip	3 × 30 s, unilateral. 2-min rest intervals for IHG-60. 4 × 2 min, unilateral, 4-min rest intervals for IHG-30 (*n* = 12 IHG-60, *n* = 13 IHG-30, *n* = 14 control)	IHG-60 142/91 mmHg IHG-30 142/89 mmHg	IHG-60 125/84 mmHg IHG-30 136/86 mmHg
Nemoto et al. (2021) [80]	Japan	8	*N* = 53 (43% female)	Pre-HTN/HTN	Yes	None	3 × per week	Handgrip	4 × 2 min, bilateral (alternating hands), 1-min rest intervals (*n* = 27 pre-set resistance value closest to participants 30% MVC, *n* = 26 control)	136.9/81.9 mmHg	134/79.5 mmHg
O’Driscoll et al. (2022) [82]	UK	52	*N* = 24 (100% male)	Pre-HTN	No	None	3 × per week	Wall squat	4 × 2 min, 2-min rest intervals (*n* = 12 95% HR_peak_, *n* = 12 control)	132.3/81.7 mmHg	121.8/73.7 mmHg
Ogbutor et al. (2019) [73]	Nigeria	24 days	*N* = 400 (45% female)	Pre-HTN	No	None	24 consecutive days	Handgrip	2 × 2 min, unilateral, 5-min rest interval (*n* = 200 30% MVC, *n* = 200 control)	133.5/87.7 mmHg	126.1/81.3 mmHg
Okamoto et al. (2020) [78]	Japan	8	*N* = 22 (59% female)	HTN	No	None	3 × per week	Handgrip	4 × 2 min, bilateral (alternating hands) 1-min rest interval (*n* = 11 30% MVC, *n* = 11 control)	156/94 mmHg	139/87 mmHg
Pagonas et al. (2017) [47]	Germany	12	*N* = 50 (60% female)	HTN	Yes	IET 1 Control 2	5 × per week	Handgrip	4 × 2 min, 1-min rest interval (*n* = 25 30% MVC, *n* = 25 sham control)	138.4/80.3 mmHg	138.4/79.6 mmHg
Palmeira et al. (2021) [71]	Brazil	12	*N* = 63 (74% female)	HTN	Yes	IET 16 Control 17	3 × per week	Handgrip	4 × 2 min, 1-min rest interval (*n* = 15 30% MVC, *n* = 16 control)	129/83 mmHg	121/79 mmHg
Punia and Kulandaivelan (2019) [74]	India	8	*N* = 40 (50% female)	HTN	Yes	None	3 × per week	Handgrip	4 × 2 min, 4-min rest intervals (*n* = 20 30% MVC, *n* = 20 control group)	144.2/92.7 mmHg	138.4/87.5 mmHg
Stiller-Moldovan et al. (2012) [46]	Canada	8	*N* = 25 (50% female)	HTN	Yes	IET 2 Control 3	3 × per week	Handgrip	4 × 2 min, 1-min rest interval (*n* = 11 30% MVC, *n* = 9 control)	112.5/84.3 mmHg	111.3/84.6 mmHg
Taylor et al. (2003) [79]	Canada	10	*N* = 17 (42% female)	HTN	Yes	None	3 × per week	Handgrip	4 × 2 min, 1-min rest intervals (*n* = 9 30% MVC, *n* = 8 control group)	156/82.3 mmHg	137/75 mmHg
Taylor et al. (2018) [29]	UK	4	*N* = 48 (100% male)	Pre-HTN	No	None	3 × per week	Wall squat	4 × 2 min, 2-min rest intervals (*n* = 24 95%HR_peak_, *n* = 24 control)	132.4/81.4 mmHg	120.1/75.4 mmHg
Wiles et al. (2009) [27]	UK	8	*N* = 33 (100% males)	NTN	No	None	3 × per week	Leg extension (bilateral)	4 × 2 min, 2-min rest intervals (*n* = 11 HI-95%HR_peak_, *n* = 11 LO-75%HR_peak_, *n* = 11 control group)	High int -121.5/68.5 mmHg	High int—116.3/65.8 mmHg
Wiles et al. (2016) [30]	UK	4	*N* = 28 (100% male)	NTN	No	None	3 × per week	Wall squat	4 × 2 min, 1-min rest interval (*n* = 14 95%HR_peak_, *n* = 14 control)	127/79 mmHg	123/76 mmHg
Yamagata and Sako (2020) [75]	Japan	8	*N* = 20 (sex unknown)	NTN	No	None	3 × per week	Handgrip	4 × 2 min, 3-min rest intervals (*n* = 10 25%MVC handgrip, *n* = 10 control group)	107.1/63.3 mmHg	102.5/60.2 mmHg

*dBP* diastolic blood pressure, *EMG* electromyography, *HTN* hypertension, *HR*_*peak*_ peak heart rate, *IET* isometric exercise training, *IHG* isometric handgrip, *mBP* mean blood pressure, *MVC* maximal voluntary contraction, *N* number, *NTN* normotension, *pre-HTN* pre-hypertension, *RCT* randomised controlled trial, *sBP* systolic blood pressure

As detailed in Table 1, there have been several meta-analytic studies collectively analysing all protocol variations of IET to provide a pooled estimate of its effects on resting BP. The first of such was performed by Owen et al. [32] and involved a limited analysis of five studies, reporting significant reductions in resting sBP and dBP by − 10.4 and − 6.7 mmHg, respectively. In the decade since, an abundance of research trials with greater methodological rigour have been performed, resulting in the publication of several larger meta-analyses including an individual patient data meta-analysis [33]. Specifically, Carlson et al. [34], Jin et al. [35], Inder et al. [36], Loaiza-Betancur and Chulvi-Medrano [37], López-Valenciano et al. [38], Smart et al. [33], Hansford et al. [39], and Edwards et al. [40] all report pooled resting sBP and dBP reductions of between 5–9 and 1–4 mmHg, respectively, in varying BP populations. The differences in effect sizes reported between particular analyses are likely owing to both the year and date in which the systematic search was performed, as well as strategic methodological differences. For example, Edwards et al. [40] strictly ensured the omission of papers published prior to the year 2000, thus excluding Wiley et al. [41] which provided early groundwork for the IET literature, but is now outdated with methodological and statistical limitations. Regardless, it is clear from all meta-analytic evidence thus far that IET is highly efficacious in the management of resting BP, with mean reductions greater than that observed following the currently recommended exercise guidelines and even greater, or at least similar, to that of standard anti-hypertensive pharmacotherapy [42].

In addition to resting BP, a smaller number of studies [43–47] have also reported the effects of IET on ambulatory blood pressure monitoring (ABPM). ABPM monitoring is recognised as a more reliable measure of BP through its increased precision, elimination of observer bias and superior predictive effectiveness in determining cardiovascular risk [48–50]. Previous work by Taylor et al. [44] observed significant reductions in 24-h ambulatory sBP and dBP by 11.8 and 5.9 mmHg, respectively, following 4 weeks of IET wall squats in unmedicated hypertensives. Additionally, Taylor et al. [44] found significant improvements in daytime and night-time sBP, mean BP (mBP) and dBP by − 13.9/− 9.4 mmHg, − 7.4/− 3.9 mmHg and − 5.6/− 4.9 mmHg. While such diurnal changes indicate enhanced BP regulation in response to daily activities during waking hours, these night-time ABPM changes are also of considerable importance given the prognostic value of nocturnal BP as a significant risk factor for cardiovascular morbidity and all-cause mortality in both normotensive and hypertensive populations [50, 51]. Specifically, as denoted by the term ‘dipping’, sleeping sBP should be > 10% lower than daytime sBP [52], a threshold to which IET may therefore be capable of providing a clinically significant contribution. Although to a lesser magnitude, similar findings have also been demonstrated in leg extension IET studies [43, 45, 53], showing significant reductions in 24-h, daytime and night-time ambulatory sBP, as well as significantly reduced morning sBP surge in both males and females [45]. Conversely, three studies investigating handgrip IET reported no change, highlighting the need for future research, particularly in unmedicated hypertensive individuals [46, 47, 54, 55].

Further to standard daytime and night-time ABPM, IET has been demonstrated to significantly improve BP variability [44]. Increased variability in BP is considered a prognostic marker for health, independent of mean BP values [56]. Previous evidence has reported significant associations between increased daytime BP variability and early development of atherosclerosis [57], target organ damage [58] and cardiovascular and stroke mortality [59]. Taylor et al. [44] found that 4 weeks of wall squat IET significantly reduced 24-h ambulatory and night-time systolic, mean and diastolic average real variability, as well as daytime systolic average real variability. Average real variability is a reliable and reproducible index for BP variability, carrying additional prognostic information for subclinical organ damage and risk of composite cardiovascular events [56].

In summary, there is evidence in support of IET as an effective anti-hypertensive intervention across a range of key BP markers including resting office BP, daytime, night-time and 24-h ambulatory, morning BP surge and BP variability. However, it is important to note that these adaptations may be specific to BP-related cardiovascular health with little-to-no evidence regarding the effectiveness of IET in improving wider traditional risk factors, such as peak aerobic capacity (VO_2_), cholesterol, or weight management.

### IET Protocol

As discussed, there are various IET protocols which have demonstrated clinically relevant reductions in resting BP, with no single uniformly accepted protocol to date. This has consequently produced a logistical gap between the current successful research findings and the practical clinical implementation of IET. As with any emerging clinical interventional strategy, establishing optimal practices with consideration of effectiveness, practicality, safety, and cost efficiency is needed.

### Mode

**Fig. 2:**
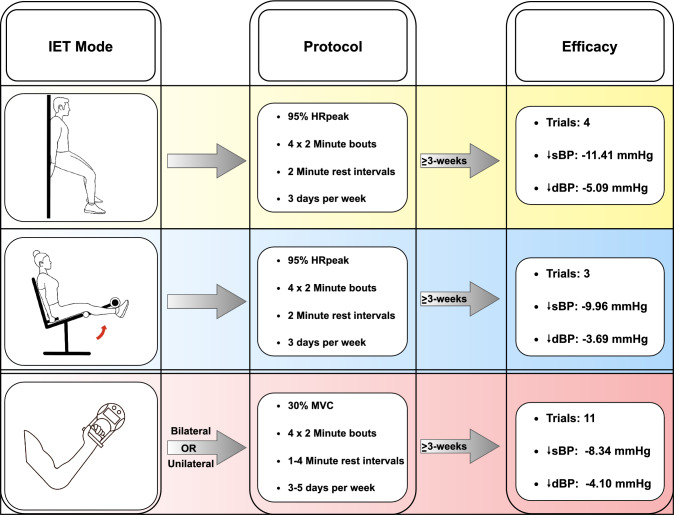
Modes of isometric exercise training. *dBP* diastolic blood pressure, *HR*_*peak*_ peak heart rate, *IET* isometric exercise training, *MVC* maximal voluntary contraction, *sBP* systolic blood pressure

Until now, there has been no robust evidence to support the superiority of one IET mode. However, considering the different stimuli, such as muscle mass, characteristics of activated muscles, and posture between wall squat, leg extension, and handgrip IET, it has been long hypothesised that clinically relevant response differences exist. The only comparative evidence of IET mode to date is provided in a recent meta-analysis, where researchers pooled the magnitude of BP change following the three primarily employed IET modes separately, and subsequently compared them as sub-groups [31]. As observed in Fig. 2, this analysis demonstrated all three modes to be effective, with sBP and dBP reductions following wall squat, leg extension, and handgrip (bilateral or unilateral) IET by − 11.41/− 5.09, − 9.96/− 3.69 and − 8.34/− 4.09 mmHg, respectively [31]. Although not statistically significant, the reduction was > 3 mmHg greater following wall squat IET than the traditionally employed handgrip mode, which is a magnitude of change considered clinically relevant [31]. This work suggests the wall squat may be the most effective form of IET despite the handgrip protocol being the most widely studied and the only protocol endorsed in any international guidelines [8]. The greater magnitude of effect with the wall squat is probably attributable to differences in the extent of recruited muscle mass and thus surface area of compressed vasculature when compared with handgrip protocols [60], while the incorporation of postural and stabilising muscles when holding the squat position may be an important distinguishing factor from leg extension IET [27]. However, these results should be interpreted with caution given the inherent limitations of such an indirect analysis and the confounding effects of differing heterogeneous participant and study characteristics. Despite the potential promise of wall squat IET, direct comparative RCTs of homogeneous populations and consistent study characteristics are required to conclude such differences.

A limitation of the primary wall squat protocol employed in most of the research is that it requires specialist incremental testing to identify an individual intensity prescription based on the squatting knee angle required to elicit a 95% peak HR response [31]. This presents logistical concerns when considering the ultimate objective of widespread clinical implementation, especially given the straightforward process of prescribing handgrip IET at 30% MVC. However, new evidence demonstrates the effectiveness of a rate of perceived exertion (RPE)-prescribed wall squat intervention which presents a more practical prescription approach [61]. Interestingly, recent acute research reported significantly higher RPE values during a single handgrip session compared with wall squat, which may carry implications for long-term adherence [62].

Ultimately, while early indirect evidence suggests that handgrip IET may not produce BP changes of the same magnitude as that of lower-body IET, it undoubtedly remains the most well-investigated mode with the strongest foundation of supporting evidence, as mentioned previously (Table 2). Handgrip IET likely constitutes the most attractive and practically implementable mode, with utility in patients with cognitive, mobility or heightened cardiovascular risk concerns. Conversely, wall squat IET may be capable of producing larger BP improvements but remains more vulnerable to implementation limitations in clinical populations and older frail adults. Leg extension IET may offer some middle ground regarding magnitude of BP change; however, it certainly suffers from limitations regarding accessibility of specialised equipment. Given the importance of training variability for exercise adherence, there is an important argument for the development of multi-modal IET routines.

### Intensity

Previous research has consistently shown intensity to be a critical training principle in the prescription of IET. Baross et al. [63] demonstrated significant BP improvements following 4 weeks of leg extension IET at 14% MVC, but found no significant change at 8%. In addition, other trials have used lower intensity IET to constitute valid sham control groups, with Carlson et al. [64] reporting significant BP reductions from 30% MVC, but not 5% MVC handgrip IET, and more recently, Decaux et al. [65] reported significant BP improvements following 95% HR_peak_ wall squat, but not 75%. As evidenced by these findings, a minimum intensity of IET is required to promote cardiovascular benefits, with 95% peak HR for squat and leg extension IET, and 30% MVC for handgrip as the most well-established intensities (Fig. 2). Although numerous trials have consistently demonstrated the effectiveness of these intensities, comparative studies investigating novel protocols of variable intensity and inter-set recovery periods are needed to truly determine the optimal IET intensity prescription for the largest magnitude of effect on BP. Javidi et al. [66] recently compared the traditional handgrip 30% MVC protocol (4 × 2 min) versus a novel 60% MVC protocol (8 × 30-s contractions), reporting significant resting BP reductions following both protocols, with significantly greater dBP reductions in the 60% MVC group. This work may provide promise for higher intensity, shorter contraction time IET protocols and ultimately highlights the importance of continued research into unexplored protocol variations.

### Frequency and Detraining

Badrov et al. [67] directly investigated the effects of two different IET training frequencies by comparing the effects of 3-times (3 ×) versus 5-times (5 ×) weekly handgrip IET sessions over an 8-week intervention. This work reported significant resting sBP reductions independently of training frequency, with no changes in dBP or mBP in either group. However, the authors reported significant mid-training (4 weeks) sBP reductions in the 5 × , but not 3 × weekly session group [67]. This finding may indicate accelerated adaptations with higher training frequency, which could have implications respective of the initial training phase and the potential for a subsequent reduction in training dosage during a maintenance phase. In the first IET study of its kind, Cohen et al. [68] recently demonstrated that BP reductions can be maintained with a single session (handgrip or wall squat) per week following a standard 3 × weekly 12-week IET programme. These findings suggest that the traditionally employed 3 × weekly IET frequency may only be necessary during the initial training phase with potential to down-titrate frequency in a maintenance phase. Despite this, there are very limited data on the implementation of a maintenance phase, with the optimisation of IET prescription remaining an area for exploration. For example, it is unclear how the potential for accelerated adaptations with greater IET session frequency in the training phase (i.e. 5 × weekly) may influence the transition from the training to maintenance phase.

Previous work from Howden et al. [69] reported a rapid detraining effect of IET where significant reductions in resting BP were mitigated within 10 days following the last IET session [69]. Early data from Wiley et al. [41] also demonstrated that BP reductions returned to baseline values following a 5-week detraining period, while Taylor et al. [44] confirmed the suitability of 3 weeks as a ‘washout’ period to establish baseline BP levels. Conversely, Baross et al. [45] found that resting and ambulatory BP reductions seen after an 8-week leg extension intervention remained significantly lower than baseline values following a further 8-week detraining period, which is a finding supported by the recent findings of Gordon et al. [70]. Evidently, the exact detraining effects regarding regression toward baseline BP values following IET are not clear and are likely influenced via training parameters such as IET mode, intensity and intervention duration. Therefore, establishing optimal IET prescription practices in respect to a minimum effective frequency dosage is not yet feasible but is critical. Regardless, the current literature is entirely centred around thrice weekly sessions, and thus the significance of any wider adjustments to training frequency is largely unknown with a clear demand for future research.

### Supervision

An important aspect of IET is its possibility to be performed with or without (home-based) supervision. Studies have utilised both home-based and supervised IET, depending on the type of isometric exercise. For instance, all studies assessing wall squat IET have utilised home-based training, whereas leg-extension IET has been conducted under supervision. Handgrip IET has demonstrated positive effects on resting BP through both home-based and supervised training [54, 55, 71, 72].

In order to compare the potential influence of home-based or supervised IET on resting BP, Farah et al. [54] conducted a randomised trial with three groups: supervised handgrip IET, home-based IET, and a control group. Handgrip IET was performed using a commercially available handgrip dynamometer. The observed results showed that only supervised handgrip IET training reduced resting and central BP in medicated hypertensive individuals. Unfortunately, the device used to perform the handgrip IET was not able to record data regarding the completion and intensity of exercise sessions. However, it is possible to speculate that the differences between supervised and home-based exercises occurred due to the absence or inadequate performance of the exercise at home.

Despite the potential simplicity and short duration of the handgrip IET protocol, a previous study [72] using a handgrip device able to record the information regarding exercise sessions observed 37% of patients with peripheral artery disease did not adequately complete the 8-week home-based training. Therefore, increased adherence monitoring and supervision (virtual or other) is necessary to ensure the effectiveness of handgrip IET when prescribing it for home-based training.

### Bilateral Versus Unilateral

Leg extension IET and handgrip IET can both be performed in either a unilateral or bilateral fashion. All studies of leg extension IET have adopted bilateral training, while the handgrip IET studies have used both unilateral [64, 66, 67, 72–75] and bilateral [46, 47, 54, 71, 76–80] approaches. In a study directly comparing unilateral and bilateral approaches, McGowan et al. [81] demonstrated that both unilateral and bilateral handgrip IET training were able to reduce the resting sBP of medicated hypertensive patients. In contrast, in a systematic review and meta-analysis conducted by Inder et al. [36], it was observed that participants undertaking unilateral handgrip IET showed a larger reduction in resting sBP than those undergoing bilateral handgrip IET (− 8.92 mmHg vs − 4.58 mmHg). No significant differences in resting dBP or mBP were observed between unilateral and bilateral IET. Therefore, while the bilateral approach may be considered superior, the unilateral approach remains open to discussion. However, the significant effects of unilateral IET on resting BP increase the possibility of using this mode of training for several populations that may be unable to perform bilateral training (e.g., post-stroke patients).

From a scientific standpoint, the implementation of unilateral exercises is intriguing as it allows for the differentiation of potential local and systemic effects of IET. Given that no study has yet examined unilateral IET with leg exercises, this remains an important gap that should be addressed in future studies.

### Duration and Adherence

As shown in Table 2, all except one IET RCT published to date are ≤ 12 weeks in duration. The only study to measure the longitudinal effects of IET is a 1-year unsupervised wall squat intervention by O’Driscoll et al. [82]. This investigation reported significant reductions in sBP, mBP and dBP by − 10.5, − 9.9 and − 8 mmHg, respectively (all *p* < 0.001). Although the study sample size was limited, this work provides the first evidence of long-term adherence to IET with 77% adherence to sessions across all participants. This finding supports the hypothesis that adherence to IET is likely to be greater than other anti-hypertensive interventions, particularly considering the well cited report that 50% of people who start an exercise programme will fail to adhere within 6 months [83]. Unfortunately, there are otherwise limited data on longitudinal adherence to IET, which remains a fundamental gap in the current literature. Data from short-term studies have reported good adherence to IET [64, 66, 73, 77, 80, 84, 85]. Palmeira et al. [71] demonstrated an immediately concerning dropout rate of 50%; however, this value was similar to that observed in the control group (48%), which indicates factors other than IET (e.g., difficulty attending exercise sessions, city traffic, etc.) were related to the poor adherence.

Given the lack of longer-term IET studies, the importance of intervention duration on the magnitude of BP reduction is not clear. Research from Millar et al. [86] effectively demonstrated linear negative trends in resting sBP and dBP over an 8-week intervention with no plateau in reductions over this timeframe. Although this work indicates greater reductions from a longer intervention duration, how this trendline may continue to adapt following an IET intervention of > 8 weeks is largely unknown. The magnitude of change found in the O’Driscoll et al. [82] longitudinal study may support a larger resting dBP effect with longer intervention duration when observationally compared with previously published identical 4-week interventions [44, 65]; however, direct research is needed. Separate meta-analysis work has offered intervention duration as a potential moderator in meta-regression analyses, but no significant effect of the number of training weeks on duration has been detected [31].

Regarding minimum duration, trials have demonstrated clinically significant reductions in resting BP following as little as 3 weeks of IET [87]. To our knowledge, no research has examined the effects of a < 3-week IET intervention; however, given the substantial changes commonly seen at 3–4 weeks, it may be pragmatic to suggest that clinically significant changes occur much before this point. With this in mind, future research is required to understand the minimum effective duration of IET before BP adaptations begin to plateau. Combined with the minimum effective frequency of IET, this information would allow for enhanced IET prescription by establishing the minimum necessary duration and frequency of training required to achieve maximal BP reductions and then subsequently maintain these changes.

Ultimately, a lack of longitudinal and minimum effective duration IET data limit the ability to generate specific efficacy and adherence inferences from the current IET duration literature. However, it can be concluded that interventions of ≥ 3 weeks to 1 year in duration can produce clinically important reductions in resting BP.

### Protocol Summary

Figure 2 presents the most well-supported IET protocol practices based on the current literature. In summary, the present evidence base supports 95% HR_peak_ wall squat and leg extension, and 30% MVC handgrip protocols, performed 3 × per week for ≥ 3 weeks, in sessions of 4 × 2-min bouts with rest intervals of 1–4 min (see ESM for full IET exercise prescription details). However, RPE protocols are emerging as more practical for the prescription of wall squat IET. RCTs are needed to truly discern the comparative efficacy and clinical utility of each IET mode; however, early, indirect work suggests the wall squat may be more efficacious than the traditionally employed handgrip IET mode, while leg extension IET is often excluded on the basis of poor practicality/accessibility. It is also important to consider that these traditionally recommended protocols of 4 × 2-min bouts at the discussed intensities have rarely been challenged and are largely rooted in original work from Wiley et al. [41] and others (see Table 2). As such, research trials such as Javidi et al. [66], which pilot new IET protocols against the traditional protocols, are to be encouraged.

## Considerations in the Interpretation of the Current Literature

### Outcome Moderators

Substantial heterogeneity and complexity in individual physiological profiles complicate the interpretation of the current IET literature. Despite consistent and reproducible mean reductions in resting BP following IET, inter-individual variance is commonly overlooked with some individuals deemed ‘non-responders’ to an IET intervention. While inter-individual variability is inherent to any anti-hypertensive treatment, identifying likely non-responders at an early stage (ideally before initiation of IET) is important in the context of personalised medicine [88]. The reasons for such inter-individual variability may be linked to differences in physical activity status, stress levels, sex, age, ethnicity, complex pre-existing comorbidities and diseases, genetics, rapid versus delayed responses and current pharmacotherapy. However, it is also important to consider the common pitfalls of non-responder identification considering random variability, as discussed by Atkinson et al. [89]. As detailed, the complexity of BP regulation is itself an inherent limitation to the underlying literature of any anti-hypertensive intervention, adding a broad layer of intricacy to the interpretation and inferences that can be made from the available IET data. The two primary confounders which are historically understood to moderate the degree to which BP changes following exercise are baseline BP and medication status.

Like pharmacological anti-hypertensive treatment, a higher baseline resting BP is generally associated with greater reductions in BP with exercise training [90]. Indeed, as shown in Table 2, the greatest BP reductions observed following IET tend to be in unmedicated hypertensive cohorts [36, 44]. This is traditionally linked to a lower threshold of BP response in hypertensives, whereas normotensive reductions may be limited by counter-regulatory processes designed to prevent BP reductions below homeostatic clinical levels (hypotension) [91]. While baseline BP may therefore constitute a significant portion of inter-study and inter-individual variance, IET has also been largely successful in multiple RCTs of normotensive cohorts, as demonstrated in a recent systematic review and meta-analysis by Loaiza-Betancur et al. [92]. This therefore supports IET in not only the treatment of HTN, but as a potential protective and preventative intervention for those with normotensive or pre-hypertensive status. However, the potency of this requires confirmation in prospective studies with long-term follow-up.

The involvement of ongoing anti-hypertensive pharmacotherapy complicates interpretation of the current IET literature. Although the individual participant data meta-analysis by Smart et al. [33] reported no evidence of a medication effect, the BP response to IET in medicated hypertensives is often lower than that seen in unmedicated hypertensives, which is likely, at least in part, attributable to overlapping mechanisms between IET and anti-hypertensive drug-induced BP reductions [40, 44, 67]. As a limited example of this, four studies have measured pre- and post-IET inflammatory biomarkers, with differing results depending on medication status, which may provide some mechanistic insight. In unmedicated pre-hypertensive or hypertensive cohorts, Taylor et al. 2018 [44], Javidi et al. [66] (IHG-30 group) and Ogbutor et al. [93] all found anti-inflammatory changes with reductions in interleukin-6 following IET, whereas Rodrigues et al. [76], who studied a medicated hypertensive population, found no change. Although these conflicting results are probably influenced by a plethora of other methodological variables, the hypothesis that IET shares common mechanistic ground with anti-hypertensive medication is certainly plausible and one that remains largely unexplored [94–97]. Thus, given the expansive number of varying anti-hypertensive drug classes, each constituting different mechanistic effects, future IET research requires participant stratification based on medication class. However, this line of research remains complicated by limited real-world clinical transferability due to the common scenario of polypharmacy. While the outlined inter-individual differences in response to IET appear pragmatically linked to the moderators discussed (baseline BP and medication status), it is important to consider the findings of a recent meta-analysis by Kelly et al. [98] who found random variability as opposed to true inter-individual response differences accounted for any differences in sBP and dBP changes following IET. Thus, while future research on inter-individual response differences to IET is undoubtedly needed, this work suggests confounding moderators are less important than traditionally believed in influencing BP responses to IET.

### Evidence Quality

There are some notable concerns regarding the methodological quality of the current IET literature. In a recent meta-analysis of RCTs [40], all trials were scored via the ‘Tool for the assEssment of Study qualiTy and reporting in Exercise’ (TESTEX), which is a 15-point quality assessment tool designed for the direct application to exercise interventional research [99]. Examining this quality assessment, the TESTEX scores of these papers primarily ranged from 7 to 10 out of a possible 15, with one study scoring 13 [64]. Although arbitrary cut-off points are debated, previous work has suggested > 12 points as ‘high quality’, 7–11 points as ‘good quality’, and < 6 points as ‘low quality’ [100, 101]. Thus, while the IET literature may be interpreted as primarily being of ‘good quality’, there are several quality points that are frequently neglected and need addressing in future IET research. In particular, the majority of research fails to blind assessors (which could be counteracted with ABPM approaches), conceal allocation from the participants eligible for inclusion (i.e. acquire consent prior to the randomisation process), perform intention-to-treat analysis where appropriate, or monitor control group activity. In some severely limited studies, there are statistically significant differences in BP at baseline, and adherence and/or session completion rates are not reported. The majority of published IET research also fails to effectively control for non-specific factors, such as the placebo effect. Controlling for non-specific factors in IET is complicated by the inability to blind participants (i.e. participants are likely to be aware that they are, or they are not, receiving IET); however, the inclusion of a sham control group who unknowingly perform IET at an intensity proven to be ineffective is a useful technique to improve general methodological rigour. This design has been effectively employed in some previous handgrip [47, 64, 102] and wall squat IET studies [65]. Combining these outlined limitations, sometimes in the form of uncontrolled and non-randomised designs, some of the weaker evidence is likely to suffer from regression toward the mean, which is a concept not exclusive to IET, but applies to all interventional research with repeated measures [103].

Regarding population numbers, studies from Ogbutor et al. [73] and Correia et al. [72] have included impressive sample sizes of 400 and 102 initially randomised participants, respectively; however, these numbers are not common across a literature that is largely limited by small sample size trials. Indeed, larger-scale research, ideally applied in a clinical setting using ABPM methods, and compared against the present exercise guidance, would be of immeasurable benefit to the current evidence landscape. In that sense, larger-scale feasibility studies, such as the IsoFIT-BP study currently applied in an NHS primary care setting, may constitute important steps forward [104].

Table 3 presents an authorship panel consensus on the certainty of evidence. We applied the constructs of the GRADE (Grading of Recommendation, Assessment, Development, and Evaluation) approach following the narrative summarisation of the information provided in this work from all studies in Table 2. This rating should provide decision makers, particularly those involved in the development of exercise guidelines, with information regarding the certainty of the current IET literature and its effects on resting blood pressure.

**Table 3:** Isometric exercise training and resting blood pressure: author panel consensus on the certainty rating of evidence

GRADE domain	Judgement				Concerns about certain domains
Methodological limitations of the studies	Many of the studies, despite randomisation, provided unclear information regarding allocation concealment. Blinding of outcome assessors was not reported in most of the studies. However, this limitation was mitigated using automatic devices for blood pressure assessments. Some studies failed to adequately account for all patients in the analysis. Selective outcome reporting and other limitations were not disclosed. Approximately 30% of the studies exhibited a high risk of bias				Serious
Indirectness	Assessment of blood pressure was performed directly using validated methods. We judged there is no evidence of indirectness				Not suspected
Imprecision	The sample size of all studies was ~ 1400 subjects. Significant reductions in systolic and diastolic blood pressure were reported in most trials with different sample sizes				Not suspected
Inconsistency	There were no variations in the direction of the estimated effect. The mean magnitude of effect for systolic blood pressure was − 8.1 mmHg (95% CI − 6.5 to − 9.7 mmHg) and for diastolic blood pressure was − 3.7 mmHg (95% CI − 2.9 to − 4.5 mmHg). The inter-study variability in the magnitude of effect is considered primarily attributable to protocol and population differences				Not suspected
Publication bias	Although unclear, we did not strongly suspect publication bias because most registered trials were published				Not suspected
**Outcome** Resting systolic and diastolic blood pressure		**Effect** Most studies show reductions in both systolic and diastolic blood pressure	**Number of participants/studies** 1424 subjects (28 randomised controlled trials)	**Certainty in the evidence** MODERATE^a^ ⊕ ⊕ ⊕ O (due to serious risk of bias)	

^a^Serious risk of bias across studies because of unclear or inadequate allocation concealment, blinding and adequately accounting for all patients in the analysis

### Comparative Research: IET Versus Exercise Guideline Recommendations

Despite a plethora of indirect analyses [88, 90, 105–107], there are limited direct data on the comparative effects of IET against traditionally recommended aerobic exercise on BP and related cardiovascular parameters. Of note, the distinctive characteristics of these exercise modes make it challenging to draw accurate comparisons, primarily due to the absence of standardised parameters in key training variables like volume and intensity. While Yoon et al. [108] found similar reductions in BP and pulse wave velocity between handgrip IET and aerobic brisk walking, the wider comparative literature appears to provide less support for IET. Preliminary training work by Ash et al. [109] supports aerobic training over handgrip IET, but is limited by a total sample size of 11 participants. Goessler et al. [55] performed a trial of greater scale, randomising 60 participants to an 8-week aerobic, IET handgrip or control group intervention. While this study found larger reductions in sBP following handgrip IET (− 5.5 mmHg; *p* < 0.01) than aerobic (− 3.9 mmHg; *p* = 0.07), dBP was significantly reduced following aerobic training (− 4.4 mmHg; *p* = 0.006) but not following IET (− 1.8 mmHg; *p* > 0.05); although it should be noted that these differences between the two modes were not statistically significant. Furthermore, aerobic training, but not IET handgrip, produced significant changes in daytime ABPM [55]. Perhaps the most notable comparative research is that by Pagonas et al. [47], who randomised 75 hypertensive patients to either 5 × weekly IET handgrip training, 5 × weekly sham handgrip training, or 3–5 × weekly aerobic exercise training. Intriguingly, while aerobic training significantly reduced resting and 24-h ambulatory sBP, this study firmly conflicts with the wider scientific literature by reporting no BP changes following IET. In response to this publication, Smart et al. [110] provided a commentary citing various methodological criticisms of the study, which were individually countered by Pagonas and Westhoff [111]. Interestingly, it should be noted that further analysis of this trial recently demonstrated significant reductions in central aortic sBP in the IET group, although no change in BP variability; however, this work was not powered for this analysis and the finding may simply reflect a type 1 error [112]. In summary, while some limitations of Pagonas et al. [47] are clearly valid, the immediate dismissal of these findings based on the presented criticisms is still a point of debate. Indeed, combined with the findings of Goessler et al. [55], this work effectively highlights the uncertainty of IET, particularly in the form of handgrip, to produce reductions that are clinically and statistically significantly greater than that seen with traditionally recommended aerobic training as was previously hypothesised. The important outcome of this correspondence and the wider literature is the need for future trials of larger sample sizes investigating the effects of IET (handgrip and wall squat) versus or in combination with aerobic training on resting and ambulatory BP. Future research may even consider reframing the research approach to IET, whereby researchers investigate non-inferiority as opposed to superiority when making such comparisons.

Recently, Fecchio et al. [85] compared the effects of 10 weeks of dynamic resistance training, handgrip IET, and their combination on resting BP in treated hypertensive men. The net reduction in systolic blood pressure (sBP) in the dynamic resistance training, IET, and combined training groups was − 8 mmHg, − 5 mmHg and − 11 mmHg, respectively, when compared with the control group. Among these, only the dynamic resistance training group showed statistically significant results and also demonstrated a net increase in peak blood flow during reactive hyperaemia, indicating improved microvascular function. However, pairwise comparisons did not reveal any significant differences among the three groups, making it uncertain whether dynamic training is superior to IET. To clarify this, future studies should not only compare dynamic training with other forms of IET but also include both hypertensive men and women in the sample.

### Sex-Based Research

IET research has been predominantly performed in a mix of male to female, or male-only cohorts (Table 2). Therefore, the efficacy of IET exclusively in females, as well as any potential sex differences are not well known.

Evidence from acute studies indicates potential sex differences in responses to a single session of IET. In female-exclusive research, O’Driscoll et al. [96] investigated the acute responses to a single IET wall-squat session, finding significant improvements in haemodynamic control, with cardiac autonomic power-spectral heart rate variability (HRV) analysis data returning to baseline readings during recovery. Interestingly, following an identical protocol in males, prior research from Taylor et al. [97] found HRV to increase following an acute bout of IET and exceed that of baseline measures. These differences are further supported by the findings of Teixeira et al. [113], who measured the acute effects of IET handgrip training and showed larger cardiac autonomic responses during recovery in males compared with females. Although unknown, there appears to be differences in the baroreflex pressor response as a primary mediator of these differences in post-IET autonomic response between males and females, with males receiving a 16-fold increase in baroreceptor sensitivity (BRS) following IET, compared with a 3.6-fold increase in females [96, 113, 114].

In training studies, Baross et al. [53] and Somani et al. [43] both found similar statistically significant reductions in systolic ABPM between men and women. Badrov et al. [115] found that resting BP, as well as the mechanistic investigation of endothelial-dependent vasodilation, did not differ between young, normotensive men and women, a finding also supported by Smart et al. [33]. Furthermore, a systematic review and narrative synthesis by Bentley et al. [116] also found no significant differences in BP reductions following a handgrip IET intervention between men and women. Interestingly, when simultaneously analysed with age, older women experienced the largest mean reductions, indicating a potential sex/age interaction in the effects of IET [116]. Although HTN remains less common in younger women than men (< 60 years of age), rates of HTN are greater in elderly women than men [117]. From 1990 to 2019, HTN rates in women have nearly doubled from 331 to 626 million people, with the age-standardised global prevalence similar to men (32 vs 34%, respectively) [118]. As such, the importance of anti-hypertensive interventions in females should not be overlooked as was traditionally the case, and therefore greater quality sex-focused IET research is encouraged [119]. Indeed, the same applies for those of different ethnic populations who are at varying degrees of predisposed cardiovascular risk [120].

### Evidence Reviewal Summary

In summary, this section aims to provide insight and contextualisation of the specific details that remain important when interpreting the broad literature and considering the direction of future research. The outlined gaps and limitations of the IET literature provide important context, but it should be noted that many of these are true for any anti-hypertensive intervention, including pharmacotherapy, which remains the most prevalent treatment option in clinical practice [18]. Despite many ongoing studies addressing these gaps, there remains a lack of large-scale clinical IET studies as the main source of evidence quality disparity between established interventions such as medical therapy and IET.

## Safety

Traditionally, IET has been commonly overlooked due to concerns over safety. These concerns have been largely centred around historical work on left ventricular and haemodynamic responses to IET [121–127]. Subsequently, a notion followed that IET induces drastic acute increases in sBP, dBP and rate pressure product (RPP), which may theoretically contraindicate such training for certain clinical populations. Indeed, the safety of IET in clinical populations with specific risks concerning acute BP changes is an imperative consideration and highlights the need for appropriate patient screening prior to the prescription of IET. For example, IET is strongly contraindicated (although on the basis of low-quality evidence) in those with connective tissue disorders (such as Marfan syndrome) [128] or thoracic aortic disease [129]. However, wider claims for the contraindication of IET in otherwise healthy hypertensive patients are unfounded and confuse clinicians and clinical exercise professionals. With respect to safety and appropriate application, the aforementioned claims are ultimately prohibitive of adoption and IET remains ignored.

Physiologically, the static nature of IET results in the compression (and occlusion in some individuals) of the active muscle vasculature, eliciting increases in cardiac output (*Q̇*) without the same magnitude of concurrent reductions in total peripheral resistance (TPR) that would generally be seen during other modes of exercise [96, 97]. Given the role of *Q̇* and TPR as the fundamentals of BP regulation (see Fig. 1), such changes would suggest an exaggerated response during IET, specifically in regard to dBP and especially in those with HTN [60, 130].

Considering that the contracting muscle mass is a crucial factor contributing to increases in BP and the subsequent compression of blood vessels, there are greater concerns regarding elevations in BP during exercises involving large muscle groups (such as squats and leg presses) compared with handgrip exercise. Aside from the wall squat, a recent modified Delphi study reported that handgrip and leg extension IET produce BP responses of > 30 mmHg sBP or 20 mmHg dBP, with smaller RPP increases compared with aerobic training [131]. Comparatively, a recent systematic review and meta-analysis [132] reported mean sBP responses of squat, leg extension and handgrip IET by + 46 mmHg, + 64 mmHg and + 33 mmHg. The differences in BP response between leg extension and handgrip IET were statistically significant.

Examining the evidence, Wiles et al. [130] measured the acute BP and RPP responses to wall squat IET in 26 hypertensive patients and reported sBP and dBP responses of 171 mmHg and 113 mmHg respectively. Importantly, the American College of Sports Medicine (ACSM) thresholds for acute BP safety are set at > 250 mmHg sBP, and > 115 mmHg dBP [133]. While no single participant recorded an sBP > 250 mmHg, dBP reached > 115 mmHg in six participants, presenting some concern. These dBP responses indicate the need for selective individualised IET prescription through the manipulation of programme variables to achieve lower BP responses. However, the relative applicability of the ACSM guidelines which were originally developed for aerobic exercise testing remains unknown, especially given that these thresholds were arbitrarily established by clinicians in the absence of data [133]. Notably, the time spent above these ACSM dBP guidelines was 4% (~ 19 s), which does not represent one single time period, but was instead spread across the training session and is therefore unlikely to elicit any significant cardiovascular risk given the short time period over which these participants were subjected to this ‘extreme’ pressor response [134]. The RPP, calculated as HR × sBP, provides an effective non-invasive index of myocardial oxygen consumption. HR responses to IET are generally much lower than that of other exercise modes and certainly do not achieve that of the ACSM exercise test attainment threshold of 85% predicted maximum HR [133]. To contextualise this, the Wiles et al. [130] population would have needed to achieve an HR response of 149 b⋅min^−1^, whereas they only observed a response of 105 b⋅min^−1^. As such, the RPP and thus myocardial oxygen consumption responses to IET are relatively small, even when compared with that of routine exercise testing in clinically vulnerable patients. For example, the highest RPP response observed in Wiles et al. [130] was 20,681 ± 2911 mmHg⋅bpm^−1^, whereas that reported in high-risk patients referred for clinical exercise testing for the evaluation of ischaemic heart disease was 27,729 ± 5018 mmHg⋅bpm^−1^ [135]. These low RPP responses are further evidenced in Carlson et al. [136] with handgrip IET. In addition, the increase in dBP is also a driver of coronary flow, which may reduce the risk of myocardial ischemia.

Intensity is another important determinant of the BP response to IE. A systematic review [132] observed that acute BP responses following IET were dependent on intensity with higher MVC handgrip IET (> 60%) eliciting more exaggerated responses than lower intensity IET. In contrast, duration did not appear a primary mediator for acute BP responses to IET. Although this review is limited by inter-study heterogeneity regarding a lack of standardisation in which BP was recorded in response to an IET contraction, overall, this work confirms that IET involving larger muscle groups, such as leg extension and wall squat IET, appear to induce a more exaggerated BP response. This therefore provides a loose framework on which clinicians can individualise IET prescription, with handgrip more likely to be suitable for those at higher risk.

To ensure the safe prescription of IET, it has been suggested that clinicians and researchers ensure patients and participants maintain frequent uninterrupted breathing throughout a contraction to avoid unintentionally performing the Valsalva manoeuvre [131]. The Valsalva manoeuvre refers to forced expiration against a closed glottis and is well known to produce significant acute increases in BP [137]. Previous work has acknowledged its importance with respect to cardiovascular risk, particularly in the context of straining for bowel movement [138]. Combined with the acute BP response to an IET contraction, incorrect breathing practices resulting in the performance of an unintentional Valsalva manoeuvre may increase BP beyond absolute contraindication thresholds, particularly in those with pre-established HTN. This communication from clinician/researcher to patient/participant is crucial as those performing IET often have a proclivity to naturally begin holding their breath during a contraction.

As an interesting caveat to the safety literature, IET is well established to produce a post-exercise hypotensive response, which generally appears in relation to the hypertensive response during IET [139–141]. Although there are no direct comparative data, the post-exercise hypotension following IET (particularly lower-body IET) appears larger than that of other exercise modes and may offer a positive counteraction to the acute BP rise that occurs during an IET contraction, particularly when combined with the chronic benefits generally observed (which may be mediated by the acute response) [141, 142].

Despite the well-recognised role of post-exercise hypotension in patients with HTN, significantly rapid decreases in BP, especially dBP, can pose risks in patients with coronary artery disease. During the diastolic phase of the cardiac cycle, blood flow supplies the cardiac muscle. Rapid decreases in dBP can reduce the flow to the myocardium, leading to transient myocardial ischemia and a consequent increase in cardiovascular risk [143]. Therefore, caution is advised for patients with coronary artery disease when performing IET involving larger muscle mass, due to an abrupt decrease in dBP [144]. Separate to cardiovascular risk, specific IET modalities can carry some less notable safety considerations. Mobility concerns, particularly in frail older patients, carry an additional risk of falls and musculoskeletal injury with the wall squat. As such, the application of wall squat IET may be limited or even contraindicated in a subgroup of patients who cannot safely hold a wall squat position due to various reasons, such as knee pathology, obesity and lack of sufficient musculoskeletal fitness or general frailty-related mobility problems. The acute hypotensive effects could also pose a risk in older participants prone to vasovagal syncope. Handgrip IET specifically may also cause hand cramping and skin irritation/discomfort with common reports of blisters and calluses from the sustained pressure. However, there may be room for improvement here in targeting the historically rigid design of handgrip dynamometers with alternative devices, such as a squeeze ball dynamometer. Acute local paraesthesia, likely attributable to metabolite build-up, is also commonly reported with IET. This most commonly presents in the form of an uncomfortable burning sensation which subsides shortly following the cessation of a contraction.

The only adverse event data of any significant scale were reported in a systematic review and meta-analysis [39]. Combining findings from pre-hypertensive IET studies to form a pooled sample size of 964 participants, Hansford et al. [39] reported a sum of eight events following IET and one in the control group. These data were subsequently extrapolated to equate to one adverse event per 28,428 bouts of IET [39]. While this contextualises the current best available evidence, the limited quality of research this finding is extracted from, combined with various other confounders such as IET mode, the health status of participants in the analysed trials and reporting bias severely impede the real clinical inferences that can be made from such analysis. It is encouraged that all future IET trials closely monitor and appropriately report adverse event data.

Overall, as summarised in the recent expert consensus Delphi study [131], the current IET data support the safety of IET in healthy people, patients with pre-HTN, stage 1 HTN, some cardiovascular diseases and peripheral artery disease. Current research is limited in providing any conclusions on the safety of IET in wider and often more complex populations, including no available evidence in patients with obesity, diabetes, or populations wherein HTN and mobility limitations are prominent. Regardless of the health status of the participating individual, the Valsalva manoeuvre should be avoided through the encouragement and teaching of appropriate breathing techniques.

## Isometric Exercise Training: Acute Physiological Responses

### Physiological Responses During Isometric Exercise

The acute physiological responses of any anti-hypertensive intervention remain the groundwork on which an understanding of long-term adaptations can be developed. Interestingly, research from Somani et al. [145] has demonstrated that the acute sBP responses to handgrip and leg extension IET can effectively predict the sBP reductions following a 10-week intervention in healthy young adults. Although the advanced cellular events underlying the acute responses are unknown, research on the systemic cardiovascular and autonomic responses to IET has advanced over several decades [146–148].

The elevation in BP during IET is primarily the result of an increase in Q̇ via a chronotropic response, while SV generally remains stable or decreases due to venous return impairment and increases in cardiac afterload [96, 97, 146, 147]. TPR has been previously suggested to provide a less active contribution to this BP rise, although the limited previous data have been largely based on transitory measures as opposed to continuous recording which may not be sufficient to effectively capture the true pressor response [130, 147, 149, 150]. Examining the current continuous data, handgrip research has demonstrated a small increase in TPR that remains above baseline when measured throughout a single contraction [151], as well as when measured throughout a 4 × 2-min protocol session [62]. Conversely, the wall squat appears to produce an initial rise in TPR, followed by a stepwise reduction throughout each interval [96, 97]. Regardless of these small response differences between modes, the conclusion is that IET does not appear to produce reductions in TPR to the same extent as seen at the onset of dynamic modes including moderate intensity continuous [142] and high-intensity interval exercise [141], which is certainly a large mediator in the BP response to IET. Mechanistically, this may be linked to the differences reported in flow mediated dilation between IET and other exercise modes within an acute setting [30, 97, 152]. In particular, IET acutely produces a mechanical response via contraction-induced compression of the relevant vasculature with resulting reactive hyperaemia and a pressure undershoot on relaxation [115]. This reactive hyperaemia subsequently enhances shear stress as a mechanical stimulus to facilitate increases in endothelial intracellular calcium via potassium channel activation, ultimately promoting endothelial NO synthase [153]. Given that such a mechanism only occurs on cessation of an IET contraction, the short rest intervals between each sustained contraction, combined with the short total duration of an IET session, may be speculated to be responsible for the lesser acute TPR reductions during IET than seen with standard exercise hyperaemia in other exercise modes (sustained vs rhythmic contractions). Without the sustained vasculature compression unique to IET, these other exercise modes may have this flow mediated dilation mechanism continuously functioning throughout a single session, resulting in more consistently elevated shear rates at the site of the local vessels, consequently promoting TPR reductions during exercise and thus a lower BP response [96, 97].

These acute BP and haemodynamic changes with IET are regulated by complex interactions between central command, the exercise pressor reflex, the arterial baroreflex and the cardiopulmonary baroreflex [154]. Specifically, an IET contraction-compression affects group III/IV afferents sensitive to mechanical and metabolic stimuli which subsequently triggers cardio-acceleratory central command responses in the form of increased sympathetic activation and concurrent parasympathetic withdrawal. Such changes in autonomic balance are implicated in the outlined haemodynamic responses to IET, including the release of catecholamines which promote positive chronotropy, inotropy, dromotropy and lucitropy via β-adrenoceptors. While the chronotropic effects are clearly evidenced through an increase in HR, the inotropic effects of this sympathetic predominance are attenuated by concurrent changes in afterload and preload [97]. This has been evidenced through a reduction in SV seen during IET [97], which differs from the response commonly observed with other modes of exercise training [141].

Investigating such cardiac autonomic changes during IET, frequency domain HRV analyses in both males [97] and females [96] have reported a stepwise reduction in the total power spectrum of HRV at the onset of an IE contraction with a greater proportion of the frequency domain within the low frequency band. Commonly, this observation would be associated with a sympathetic response during IET; however, methodological limitations in the interpretation of low frequency as an accurate measure of sympathetic tone have been commonly presented, with Goldstein et al. [155] suggesting that low frequency serves as an index of baroreflex function rather than sympathetic tone [141]. Conversely, BRS appears to significantly decrease during IET [96, 97], which is associated with the withdrawal of parasympathetic activation as previously measured via high frequency HRV. This significant reduction in BRS during IET represents the resetting of the baroreceptors to allow for a higher BP and HR as demanded by the exercise; a response commonly seen in other forms of exercise [141, 156]. Irrespective of the confliction surrounding the validity of frequency domain HRV, evidence from differing methodological approaches such as muscle sympathetic nerve activity (MSNA) and plasma noradrenaline spillover support a sympathetic response during IE [154, 157–159].

Broadly, the acute BP responses during IET appear proportional to the relative intensity and duration of contraction, as well as important programme variables such as rest period between each contraction, number of contractions per session and of course, the IET mode performed [130, 160, 161]. Recent comparative research has shown wall squat IET to elicit larger bout-to-bout elevations in BP than that of handgrip as a result of a greater HR and thus *Q̇* response [62]. Despite variance in the magnitude of change [162], the general trend of these during-IET BP, haemodynamic and autonomic changes appear consistent across males and females performing either handgrip or wall squat IET [62, 96, 97].

### Physiological Responses Post-isometric Exercise

**Fig. 3:**
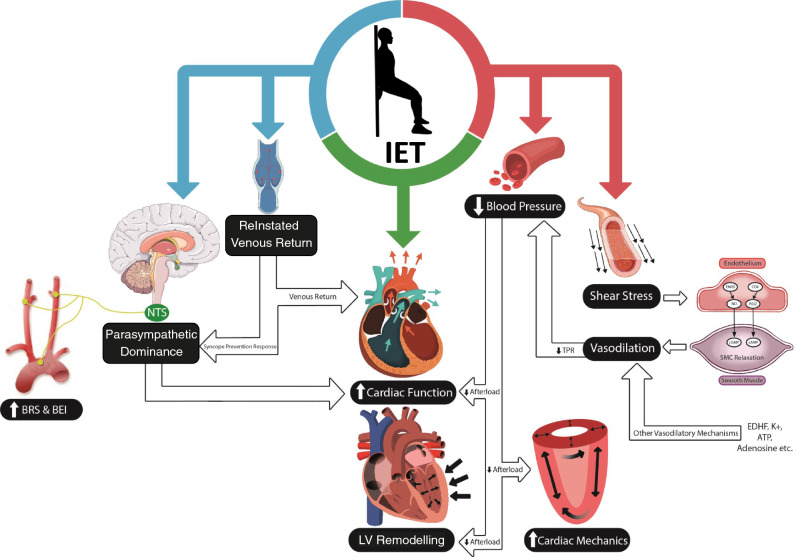
Acute physiological responses post-isometric exercise. *ATP* adenosine triphosphate, *BEI* baroreflex effectiveness index, *BRS* baroreflex sensitivity, *EDHF* endothelium-derived hyperpolarising factor, *IET* isometric exercise training, *K* potassium, *LV* left ventricular, *NTS* nucleus tractus solitarius, *TPR* total peripheral resistance

The acute physiological responses typically seen after IE are depicted in Fig. 3. As observed, cessation of an IE contraction allows for rapid reperfusion of blood to the previously compressed vasculature in a period of post-IET reactive hyperaemia [163]. This reactive hyperaemia results in an elevated shear rate against the localised endothelial lining to stimulate the secretion of flow-induced vasoactive substances such as NO, prostaglandins, potassium, adenosine triphosphate and other important vasodilatory mechanisms that are not well established in the context of IE [164, 165]. Simultaneously, there are fundamental autonomic and baroreflex changes immediately following IE, with shifts towards parasympathetic predominance, sympathetic withdrawal, and concurrent increases in BRS [243, 244]. Associated with this vagal action, the increase in BRS during recovery suggests a post-exercise resetting of the baroreceptors as HR and subsequently blood pressure begin to reduce [166, 167]. As previously reported [97, 144], venous return is increased post-IE, resulting in a reinstated preload. Combined with a reduction in afterload, this increase in preload contributes towards acute cardiac functional, structural and mechanical improvements via the Frank-Starling law, ultimately increasing SV and *Q̇* to slightly above baseline [166, 167]. Specifically, IET elicits statistically significant acute improvements in cardiac systolic and diastolic function, relative wall thickness, fractional shortening, and cardiac mechanics (global longitudinal strain and untwisting) [144]. With *Q̇* remaining near baseline irrespective of these acute cardiac adaptations, the post-IET hypotensive changes must be predominantly driven by TPR reductions.

Acute post-IET hypotensive effects are generally supported, particularly for the wall squat, with previous research showing acute statistically significant sBP reductions below baseline by − 23.2 mmHg in males, and − 17.3 mmHg in females [97] [216–218]. However, the post-exercise hypotensive effects of handgrip protocols have been less clear, with some studies even reporting no change in cohorts of older women [168] and medicated hypertensive patients [169, 170]. Certainly, the acute post-IET BP responses reported in different trials are dependent on various methodological factors. Those studies measuring more immediate values (≤ 10 min) tend to report larger reductions [62, 96, 97] than more delayed measures (≥ 30 min) [169, 170]. Indeed, some work suggests that a single acute IET handgrip bout may produce immediate BP reductions, but without any sustained post-exercise hypotension as determined through ABPM [109, 171]. IET mode appears to be an obvious moderator, with Swift et al. [62] demonstrating greater post-exercise hypotensive responses following wall squat compared with handgrip IET. Interestingly, while the wall squat group produced the largest reductions 10 min following IE with steady attenuation of this response at 1 h, the handgrip group demonstrated the greatest BP reductions at 1 h following IE, suggesting a more sustained response. Undoubtedly, future research with standardised and comparable methodologies are needed to comprehensively understand the acute BP responses and subsequently sustained post-exercise hypotension following IE amongst different participant populations and IET protocols. Regardless of the conflicting literature findings, it is a common theme that greater exercise BP and haemodynamic responses during IE often produce the greatest post-IE changes, highlighting the need to generate a sufficient acute stimulus for a response. This may serve as evidence for the integral role of IET intensity and protocol prescription in eliciting the necessary acute responses which may subsequently translate into the desired chronic adaptations.

Similar to the responses during IE, the post-IE responses and underlying mechanisms are dependent on a variety of moderators that remain heterogeneous from study to study. These may include IET mode, protocol, age, sex, ethnicity, disease, medication and methodology, which should all be considered independently and collectively as factors when implementing IET.

## Isometric Exercise Training: Chronic Physiological Mechanisms

### Mechanistic Overview

As demonstrated in Fig. 1, BP is fundamentally regulated by Q̇ and TPR. Therefore, any acute or chronic BP changes following IET must involve either or both of these integral factors. The advanced details regarding these underlying mechanisms, particularly on a cellular level, are still largely unknown with most research based on small-scale work. An overlap in mechanisms between IET and anti-hypertensive medical therapy-induced BP reductions complicates clinical interpretation of this literature.

The largest-scale mechanistic work to date is a systematic review and meta-analysis [31] of all IET RCTs in which BP changes were reported alongside at least one mechanistic variable. With a pooled analysis of 18 RCTs (628 participants), this work found a statistically significant reduction in resting HR by − 1.55 b⋅min^−1^ (95% CI − 0.14 to 2.96; *p* = 0.031), concurrent with a statistically significant increase in SV by 6.35 mL (95% CI 0.35–12.60; *p* = 0.038). There was consequently no change in Q̇ across these studies. Conversely, TPR significantly decreased by − 100.38 dyne⋅s^−1^⋅cm^5^ (95% CI − 14.16 to − 186.61; *p* = 0.023) alongside significant improvements in the low frequency to high frequency HRV by − 0.41 (95% CI − 0.09 to − 0.73; *p* = 0.013) and BRS by 7.43 ms⋅mmHg^−1^ (95% CI 4.29–10.57; *p* < 0.001). Ultimately, this analysis concluded a reduction in TPR, potentially mediated through enhanced autonomic vasomotor control, to be primarily responsible for the observed reductions in BP with IET [31]. While the findings of this work provide a strong base for understanding the gross mechanistic adaptations to IET, performing a pooled analysis of several heterogeneous studies that are not statistically powered to examine these mechanisms as the primary outcome is inherently limited. Furthermore, this work is restricted to gross-level changes without the scope to draw on more advanced fine-level physiological adaptations. Therefore, an in-depth exploration of each mechanistic domain is necessary to effectively understand and encapsulate the wider mechanistic literature.

### Central Adaptations: Stroke Volume

There are several documented central adaptations to an IET intervention. Similar to the outlined acute cardiac responses, recent work has demonstrated statistically and clinically significant improvements in cardiac structure, function and mechanics following 4 weeks of home-based wall squat IET [172]. In particular, wall squat IET improved key measures of systolic performance such as global longitudinal strain and left ventricular ejection fraction, as well as markers of diastolic function including tissue doppler parameters and estimated filling pressures [172]. These cardiac parameters are almost all understood as ‘load-dependent’ parameters, suggesting that such adaptations are primarily a basic consequence of the observed simultaneous reduction in resting BP and thus cardiac afterload. This is further supported by improvements in cardiac time intervals with the same intervention [173], highlighting the favourable LV and aortic pressure–volume changes that occur with a reduction in BP from IET. Interestingly, this work also found independent improvements in measures of global myocardial work (global wasted work and global work efficiency), which represents a novel approach to assessing cardiac function by incorporating afterload into its algorithm to generate less load-dependent indices [172, 174]. Inter-linked with these cardiac adaptations is the role of ventricular filling and preload, with IET inducing significant improvements in end-diastolic volume, likely due to improvements in LV relaxation [172] which has drawn speculation on the potential role of IET in heart failure with preserved ejection fraction in an ongoing clinical trial [175]. This increase in preload has implications relating to the Frank-Starling law as well as LV stretch-induced NO stimulation relating to cardiac excitation–contraction coupling [176–178]. Collectively, these afterload- and preload-dependent cardiac adaptations are likely responsible for small increases in SV often (but not always) seen following IET [31].

### Central Adaptations: Resting Heart Rate

Although some research has evidenced statistically significant and mechanistically relevant reductions [30, 63, 179], resting HR is not traditionally understood as a primary mediator of BP changes following IET, especially given that several studies have reported substantial BP reductions with little or no change in resting HR [44, 64, 65, 67, 77, 78, 82, 94, 180, 181]. Considering the limited diversity in recruited populations and small sample sizes often used in these IET trials, combined with the complexity and potential for measurement error if not appropriately controlled for in methodological design (e.g., female menstrual cycle), it is not clear if a change in resting HR has a different active mechanistic contribution towards BP reductions in particular populations. For example, moderator analysis from a recent systematic review and meta-analysis [40] suggested that studies including medicated participants observed significantly larger resting HR reductions following IET than unmedicated. With the effectiveness of HR-modulating anti-hypertensive pharmacotherapy such as β-blockers, it is possible that resting HR may assume a more central position as a mechanistic co-ordinator in different sub-populations, particularly in traditional essential HTN patients with autonomic dysfunction and thus an elevated baseline resting HR. It is likely that a reduction in resting HR following IET is mediated via autonomic vagal tone improvements [44, 94, 181].

### Central Adaptations: Cardiac Output

With small improvements or no change seen in both SV and resting HR, Q̇ tends to remain stable after an IET intervention. This is demonstrated after both short term and longitudinal IET interventions, with a recent IET intervention evidencing no change in *Q̇*, but significant reductions in resting BP and TPR [82]. Despite this, some studies [30] have found significant reductions in resting *Q̇* without changes in TPR. The relevance of this finding in the context of the wider literature is not known, but it is worth noting that this research was performed in rather young, physically active, normotensive participants who have a different physiological and risk factor profile to those studies recruiting older patients with essential HTN.

### Cardiac Autonomic Adaptations

Autonomic dysfunction, as characterised by an impaired sympathovagal balance, has been long implicated in the multifactorial aetiology of HTN [182–184]. Numerous studies have demonstrated improvements in autonomic cardiovascular control following an IET intervention as measured by frequency-domain HRV metrics [44, 65, 94] and non-linear heart rate complexity (sample entropy) [181]. The theoretical translation of IET-induced autonomic adaptations into clinically relevant reductions in BP is likely seen through the complex interacting effects of several BP modulating influences, such as vascular vasomotor activity and possible effects on the renin-angiotensin aldosterone system, as illustrated in Fig. 1 [183, 185].

Two studies [44, 65] have evidenced improvements in cardiac BRS after 4 weeks of wall squat IET, although Decaux et al. [65] was not powered to show a significant difference. The baroreflex is a vagally mediated integral regulator of BP, with an increase in BRS serving to improve both BP and BP variability [44]. However, these measures are local to cardiac BRS and do not reflect the sympathetic arm of the baroreflex, which is responsible for controlling the vasculature and subsequently BP to a larger degree [262, 264]. Additionally, there are limited data to support the role of BRS in long-term BP control [186]. Despite some promising findings, there remains a substantial degree of confliction amongst IET trials measuring cardiovascular autonomic modulation [27, 102]. Studies by Wiles et al. [27], Ray and Carrasco et al. [102] and Badrov et al. [67] have all observed significant BP reductions with no change in cardiac autonomic metrics, as summarised in a small-scale handgrip IET meta-analysis by Farah et al. [187]. Intriguingly, these studies have primarily incorporated younger, normotensive, active populations and in theory, hypertensive, older, inactive cohorts are much more likely to have an impaired baseline sympathovagal balance and therefore a greater capacity for adaptation in such a domain [264]. It is plausible that some of the current confliction in autonomic findings in the IET literature may be explained, at least in part, by participant characteristic differences. For example, a subset of studies that include medicated HTN patients have shown no improvements in HRV measures of cardiac autonomic function following IET, as demonstrated in Millar et al. [181], Farah et al. [54], Correia et al. [72] and Palmeira et al. [71]. As such, it may be speculated that improvements in autonomic modulation are most distinguished in those with poorly controlled BP, which may help mechanistically explain the larger magnitude of reduction often seen in these populations. Alternatively, in younger normotensive individuals and those with well controlled medicated HTN, it is likely that alternative adaptations are predominantly responsible for the reductions in BP.

It is also important to note that this line of research may also be complicated by the application of different autonomic measures across these studies, which may have heterogeneous sensitivities in detecting more subtle changes. For example, in those studies that only demonstrated modest BP adaptations following IET, traditional HRV parameters may be too insensitive to detect such small changes in cardiac autonomic modulation, as supported by Millar et al. [181]. Furthermore, methodological concerns regarding insufficiently powered sample sizes and the overall robustness of HRV as an indirect measure of cardiac autonomic modulation remain. There also remains substantial inherent bias regarding the effects of HR change on HRV, which has not been adequately addressed in any IET work to date.

In summary, the results of studies examining change in autonomic function have conflicting results. Although unclear, improvements may be dependent on participant characteristics, with more pronounced improvements in uncontrolled HTN and a notable absence of such adaptations following IET in young normotensive individuals or those with controlled HTN. This domain of literature is complicated by differing measures and sub-measures of autonomic modulation and methodological limitations. Ultimately, at best, cardiac autonomic measures represent a surrogate of autonomic nervous system modulation as a plausibly implicated mechanistic change following IET. However, cardiac autonomic parameters such as HRV cannot provide any further detail on whether BP changes are *Q̇* or TPR driven.

### Vascular Adaptations

Given the frequent reports of no change in *Q̇*, it is likely that reductions in BP following IET are primarily mediated by vascular changes. In support of this, studies applying non-invasive measures of TPR have demonstrated significant improvements after an IET intervention on several occasions [44, 65, 82], as highlighted in a recent meta-analysis [31]. Despite this, the current literature has not comprehensively addressed the degree to which these vascular changes following IET are locally regulated via endothelial-dependent mechanisms, or systemically modulated via structural remodelling and/or functional adaptations in autonomic vasomotor control.

### Vascular Adaptations: Local Endothelium-Dependent Mechanisms

Improvements in conduit artery endothelial function are mechanistically plausible as a consequence of repeated bouts of acute endothelial stimulation with IET. As a gold-standard non-invasive measure of endothelial function, previous studies have investigated the effects of IET on flow-mediated dilation, demonstrating mostly positive results. Early work from McGowan and colleagues [81, 188] reported improvements in endothelium-dependent, but not endothelium-independent, vasodilation following 8 weeks of handgrip IET in medicated HTN participants. While some discrepancies exist [54, 189], these improvements in endothelial function have been more recently replicated in medicated, (majority hypertensive) peripheral artery disease patients [72] and unmedicated HTN patients [66]. However, as evidenced in the work from McGowan et al. [81, 188], flow-mediated dilation improvements after IET only occur in the trained arm, suggesting that such endothelium-dependent vasodilation adaptations are limited to the locally stimulated vasculature [81, 188]. This is further supported in the findings of Baross et al. [63] who found significant improvements in femoral artery blood flow, blood velocity, diameter and vascular conductance after 8 weeks of leg extension IET, but no changes in brachial artery measures. Recent knee extension resistance training also echoes these findings with localised improvements in femoral artery endothelial function but citing that the mechanical stimulus (increase in *Q̇* and in turn, shear rate) was not sufficient to trigger adaptations in the brachial artery [190]. Finally, a meta-analysis of 23 trials demonstrated that both dynamic resistance training and IET increase flow-mediated dilation in healthy individuals and subjects with cardiovascular and metabolic diseases, with no difference between the types of exercise [191].

It is important to consider that, despite the well-established notion that BP is regulated at the level of the resistance vessels [67], most IET trials investigating functional vascular changes have been applied at the conduit vessel level (brachial or femoral artery measures). Considering this, Badrov et al. [67] investigated the effects of an 8-week handgrip IET intervention on forearm reactive hyperaemic blood flow in normotensive participants, demonstrating significant increases by 42% and 57% in 3 × and 5 × weekly IET groups. Combined with the earlier findings of McGowan et al. [192] that evidenced a larger post-reactive hyperaemic response, these data support the effects of IET on resistance vessel function further to that of conduit vessel function. However, it should be noted that, despite a significant reduction in resting BP mid-intervention (4 weeks), changes in forearm blood flow trended towards an increase but were not statistically significant [67].

A promising study abstract was published which reported findings of improvements in resistance vessel endothelial function in the contralateral arm following a 4-week handgrip IET intervention [193]. However, there is otherwise limited evidence to suggest these adaptations extend beyond the locally trained vasculature [272]. The localisation of these adaptations may also help explain the difference in magnitude of BP reduction between handgrip and lower body IET, with wall squat and leg extension protocols involving larger muscle groups and thus a greater degree of functionally adapted vasculature.

Overall, IET is associated with improvements in local conduit and resistance vessel endothelial function; however, the vascular mechanisms responsible for reductions in BP following IET are likely multi-factorial and the exact contribution of these local functional adaptations remains unclear. The roles of population, medication status and a possible ceiling effect are also unknown.

### Vascular Adaptations: Neural Vasomotor Control

Changes in the neural regulation of vascular tone may constitute an important mechanistic pathway for BP reductions following IET, as supported in the findings of a recent meta-analysis [31]. Ray and Carrasco et al. [102] measured MSNA, which is a direct measure of vasoconstrictor neural activity to skeletal muscle, finding no significant change in MSNA following handgrip IET. While further research into MSNA following IET is warranted, it may be considered a measure that primarily contributes to short-term BP modulation as opposed to long-term control [194]. Despite confliction, current data support a reduction of vasomotor tone following IET.

### Vascular Adaptations: Structural Vascular Remodelling

It has been suggested that vascular adaptations following IET are biphasic, with shorter duration interventions associated with the aforementioned functional changes, whereas longer duration IET interventions have been linked to structural vascular remodelling. Following this concept, the majority of IET data, including the discussed mechanistic meta-analysis [31], only reflect short-term functional changes with limited information on longitudinally stimulated structural mechanistic adaptations. Baross et al. [53] and Gordon et al. [70] have both recently evidenced a sustained effect of IET on BP after a detraining period following 8 and 12 weeks of IET, respectively. Given that a rapid detraining effect would be expected if the mechanisms driving such BP reductions were purely functional, this work may therefore imply some degree of sustained structural [70] adaptation. Furthermore, a recent study [82] documented the longest IET intervention to date of 1 year and reported a significantly reduced TPR as the primary mechanism for the maintained BP reductions. While the mechanisms responsible for these TPR reductions were not further explored, such intervention duration warrants the consideration of structural changes.

Unfortunately, little work to date has directly investigated vessel structure following longitudinal IET. Previous work from Baross et al. [63] found a significant increase in femoral mean arterial diameter following an 8-week leg extension IET intervention, but no change in brachial diameter. Given the previously demonstrated localisation of these functional changes, these adaptations likely reflect endothelial-mediated dilatory adaptations, rather than evidence of a sustained structural enlargement [195]. Other studies measuring conduit vessel diameter following short-term IET observed little or no change [72, 76, 153].

Several studies have investigated the effects of IET on arterial stiffness, with mixed results. Okamoto et al. [78] included a cohort of unmedicated physically inactive participants and reported improvements in augmentation index following 8 weeks of IET, a finding the authors have recently reproduced [196]. Differently, in a group of medicated hypertensives, Farah et al. [54] found no significant pulse wave velocity or augmentation index changes after 12 weeks of IET. More recently, Correia et al. [72] recruited a cohort of medicated patients with peripheral artery disease and also reported no effect on pulse wave velocity or augmentation index from an 8-week IET intervention. However, the vascular abnormalities and arterial stiffness in patients with peripheral artery disease complicate the possible inferences made from such data. Despite one finding of central (but not peripheral) pulse wave velocity improvements in medicated participants [76], the general literature tends to suggest a confounding effect of baseline medication status on the efficacy of IET in improving arterial stiffness. It is also important to consider that most studies employ standard carotid-femoral pulse-wave velocity measures as opposed to the localised limb.

While the underlying mechanisms driving arterial stiffness improvements are unclear, it has been previously hypothesised that structural changes through an improvement in the synthesis and degradation of collagen and elastin, which remain the key scaffolding proteins of arterial structure and stiffness [197], are implicated. This hypothesis is supported by evidence of chronic enhancements in clinical markers of inflammation and oxidative stress following an IET intervention [44, 66]. However, to date there are no longitudinal (> 12 weeks) data on measures of arterial stiffness with IET. Therefore, the current literature is likely reflective of functional changes underlying arterial stiffness improvements following IET, with any discussion of systemic structural vascular remodelling remaining mostly speculative.

Overall, as evidenced in the recent large-scale mechanistic meta-analysis recently published [31], it is important to note that data on vascular adaptations following IET, be it functional or structural, remain limited. Although it is clear that reduced TPR is fundamental to the BP reductions seen with IET, the exact mechanistic underpinnings of this are inconclusive with a likely dependency on population/patient characteristics, IET mode and intervention duration. Functional improvements in endothelial function and vasomotor tone are mechanistically implicated, while hypotheses of structural vascular remodelling are still speculative.

#### Inflammation and Oxidative Stress

A small number of IET studies have provided small-scale data on biomarkers which may provide further information on the physiological underpinnings of BP responses to IET. Inflammation and oxidative stress have been long implicated in the pathophysiology of HTN and remain key mechanistic areas of interest [198]. Changes in inflammation and oxidative stress following IET are likely attributable to the discussed improvements in NO-dependent conduit and resistance vessel vasodilation by this strong anti-inflammatory and antioxidant molecule [199]. Indeed, a recent acute study demonstrated significant increases in NO and antioxidant defence after a session of IE bench and leg press [200].

As outlined above, IET is capable of improving inflammatory markers, particularly IL-6 [44, 66, 73], although these adaptations appear dependent on medication status [76]. Recent pilot research from Bennett et al. [201] is the first to report inflammatory changes to be linked to sBP changes following 6 weeks of IET. Regarding oxidative stress, an early uncontrolled trial by Peters et al. [202] reported reductions in aerobic exercise-induced oxygen-centred radicals and improvements in the ratio of whole blood glutathione to oxidised glutathione, simultaneous to significant reductions in sBP after 6 weeks of IET in unmedicated HTN. More recently, Javidi et al. [66] assessed both inflammatory and oxidative stress responses to an 8-week 30% or 60% MVC handgrip intervention. This work found improvements in endothelin-1 (although this has limited inferences) and carbonyl protein following handgrip IET at 60% MVC, but not 30%, with no improvements in malondialdehyde, or total antioxidant capacity at either intensity. Conversely, 30% MVC but not 60% MVC IET elicited consistent anti-inflammatory effects with reductions in IL-6 and tumour necrosis factor-α. As both intensities produced significant BP reductions concurrent with inconsistent changes in oxidative stress and inflammation, Javidi et al. [66] concluded that IET may produce BP reductions independently of antioxidant and anti-inflammatory changes. As is the case for almost all IET mechanistic data, these studies are powered to detect BP changes as the primary outcome so it is possible that a lack of consistent changes in the selected markers of inflammation and oxidative stress is the consequence of limited statistical power. Thus, while IET is capable of producing favourable changes in inflammation and oxidative stress, future well-powered research, ideally of longer intervention duration, is needed to ascertain the mechanistic importance of these changes in regard to the adjacent reductions in BP.

#### Mechanistic Summary

In summary, the mechanistic changes driving reductions in BP following IET are complicated and still largely unclear. With little or no change in *Q̇*, it appears that adaptations to the vascular system, which ultimately produce a reduction in TPR, are key. Of these, IET has been previously associated with improvements in locally regulated conduit and resistance vessel endothelial-dependent vasodilation, functional adaptations in autonomic vasomotor control, and systemically modulated structural vascular remodelling. While it is probable that multiple vascular mechanistic pathways are involved simultaneously, this review highlights the short intervention duration of studies currently available, thereby suggesting functional improvements in localised endothelial-dependent vasodilation and vasomotor tone are most likely implicated, while hypotheses of structural vascular remodelling are still speculative with longitudinal mechanistic data needed.

**Fig. 4:**
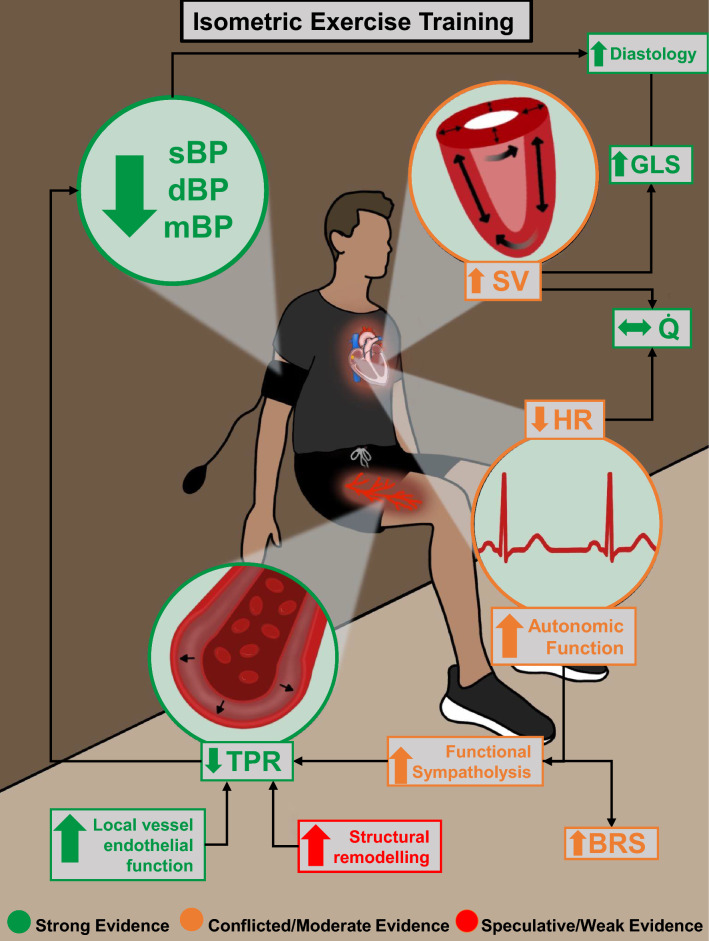
Chronic mechanistic changes seen with isometric exercise training. *BRS* baroreflex sensitivity, *dBP* diastolic blood pressure, *GLS* global longitudinal strain, *HR* heart rate, *mBP* mean blood pressure, *Q̇* cardiac output, *sBP* systolic blood pressure, *SV* stroke volume, *TPR* total peripheral resistance

Although this section provides an overview of the literature findings, it is not clear if these mechanistic changes are the same amongst differing populations. Certainly, inter-individual and inter-population variation in mechanistic findings complicates our current understanding, with particular consideration of baseline BP and medication status. Future well-powered research with a mechanistic focus is likely to help clarify the current confusion and uncertainty in the literature. Finally, the effect of IET on other potentially important external mechanistic domains, including renin-angiotensin aldosterone system factors and other mediators of vasodilation such as endothelin, prostaglandin, vasopressin and brain natriuretic peptide, remain undetermined and thus have not been discussed. This review and the outlined gaps in the literature should be incorporated into the strategic planning of future mechanistic investigatory research. Figure 4 illustrates our current understanding of the mechanistic adaptations following IET.

## Conclusion

This review summarises the potential role of IET as an anti-hypertensive intervention with consideration of its efficacy, acute cardiovascular stimulus, and physiological mechanistic underpinnings. Data from prospective RCTs and meta-analyses indicate IET is capable of producing reductions greater than that observed following the currently recommended exercise guidelines and possibly even greater, or at least similar to that of standard anti-hypertensive monotherapy. The current evidence primarily supports protocols of 95% HR_peak_ for wall squat and leg extension, and 30% MVC for handgrip, performed three or more times per week for ≥ 3 weeks, in sessions of 4 × 2-min bouts with rest intervals of 1–4 min. Handgrip protocols in particular have received endorsement in previous international guidelines [8].

It is important to acknowledge that these protocols have rarely been challenged and thus research piloting novel IET protocols are encouraged. The effectiveness of IET may be dependent on the magnitude of muscle mass recruited, with wall squat and leg extension IET appearing more effective than the traditionally employed handgrip mode. However, the convenience and reach of handgrip IET in populations with mobility or risk limitations cannot be ignored, nor the anti-hypertensive effects overstated. IET appears safe in patients with pre-HTN, stage 1 HTN, some cardiovascular diseases and peripheral artery disease, although the current literature is limited in providing any conclusions on the safety of IET in wider and often more complex populations, such as those with aortic pathology or connective tissue disorders. The acute haemodynamic and autonomic responses to IE are largely understood and may be useful in predicting chronic changes and providing advanced mechanistic insight. Chronically, the mechanisms driving changes in BP following an IET intervention are still elusive, but appear primarily dependent on changes in TPR. Vascular changes in locally regulated conduit and resistance vessel endothelial-dependent vasodilation and functional adaptations in autonomic vasomotor control are implicated, while a lack of longitudinal data limits the possibility to generate accurate inferences regarding the mechanistic role of systemically modulated structural vascular remodelling. The mechanisms responsible for BP reductions with IET are almost certainly multi-factorial and narrowing the exact contribution of each potential pathway is complicated by an underdeveloped body of evidence and inter-study variation.

Given that IET has only been investigated as an anti-hypertensive intervention for a relatively short period of time, there are still several domains within the literature that require attention. Importantly, IET research is still mostly limited to small sample sizes and thus there remains a need for large-scale RCTs ideally applied in a clinical setting and compared against the present exercise guideline recommendations. Indeed, a lack of large-scale clinical IET studies remains the main source of evidence quality disparity between established interventions such as medical therapy, traditional exercise, and IET. Future investigation into inter-individual and inter-population heterogeneity regarding BP response to IET is also needed, with particular emphasis on participant stratification based on sex, baseline BP and active medical therapy. Furthermore, adequately powered studies investigating important mechanistic variables, ideally of longitudinal intervention designs, are warranted. The methodological rigour of future IET trials may benefit from an improved implementation of assessor blinding, appropriate concealing of group allocation from participants eligible for inclusion, monitoring of control group activity and controlling for non-specific factors such as the placebo effect (i.e., employing a sham control protocol).

### Future Research Direction: Key Considerations

The following take-home points represent summarised areas of research design and direction, identified through this review and as general recommendations by the author team, that are most in need of future investigatory attention.

Design:

- RCTs with adequately powered sizes are needed to generate the same quality of evidence as that of traditional training modes.
- Multi-centre RCTs applied in a clinical setting through personalised exercise referral to match ethnic and sociodemographic diversity.
- RCTs sufficiently powered to investigate underlying mechanistic variables behind the success of IET in reducing resting BP.
- Longitudinal IET study designs with consideration of clinical and economic outcomes.
- Due to confounding variables with RCTs, wait-list design research trials where participants represent as their own control should be explored. Greater focus on methodological rigour through control group monitoring, assessor blinding, controlling for non-specific factors and minimising measurement error through a prioritisation of 24-h ambulatory, awake daytime and asleep night-time measures is needed.

Direction:

- Further direct research comparing IET in combination with and against traditional exercise guideline recommendations.
- Investigation into inter-individual and inter-population heterogeneous BP responses to IET.
- Research into adoption and adherence and individualised exercise prescription of IET.
- Exploration of IET in differing clinical conditionsand ethnic populations; generation of female-specific data.
- Investigation of the effects of IET on cerebrovascular health.
- Investigation of effectiveness of IET prior to initiation of pharmacotherapy or in combination with medical therapy and in those with resistant HTN and other BP phenotypes.
- Quantifying health-system level feasibility (e.g., patient and physician burden).
- Understanding the impact of timing of IET therapy (morning vs evening) on non-dippers, morning surge, and sleep.
